# A revision of the genus *Teleopsis* Rondani (Diptera, Diopsidae) in Sri Lanka with descriptions of two new species and a review of the other stalk-eyed flies from the island

**DOI:** 10.3897/zookeys.946.53108

**Published:** 2020-07-06

**Authors:** Hans R. Feijen, Cobi Feijen

**Affiliations:** 1 Naturalis Biodiversity Center, P. O. Box 9517, 2300 RA Leiden, The Netherlands Naturalis Biodiversity Center Leiden Netherlands

**Keywords:** *
Cyrtodiopsis
*, *
Diopsis
*, *
Sphyracephala
*, sexual dimorphism, stalk-eyed flies, *
Teleopsis
*

## Abstract

The literature on Sri Lankan Diopsidae is reviewed. Eight Diopsidae are now known to occur in Sri Lanka, five species in the genus *Teleopsis* and one species each in the genera *Sphyracephala*, *Diopsis*, and *Cyrtodiopsis*. The presence of *Cyrtodiopsis* requires confirmation to exclude the possibility of mislabelling. All five *Teleopsis* species are endemic, as are the *Diopsis* species and probably the *Cyrtodiopsis* species. Only *Sphyracephala bipunctipennis* Senior-White has a larger distribution as it also occurs in India. A key is presented for the Diopsidae of Sri Lanka. Three *Teleopsis* species were already known to occur in Sri Lanka: *T. ferruginea* Röder, *T. krombeini* Feijen and *T. maculata* Feijen. These species form the *T. ferruginea* species group. Two new species are now described for this group: *Teleopsis neglecta***sp. nov.** and *Teleopsis sorora***sp. nov.***Teleopsis ferruginea* is redescribed, as an earlier redescription turned out to be based on a series of specimens of its sister species *T. sorora***sp. nov.** The other three Diopsidae of Sri Lanka are listed and illustrated. Allometric aspects of the five *Teleopsis* species are discussed. Three *Teleopsis* species are sexually dimorphic with regard to eye span, while two species are monomorphic. It is assumed that sexual dimorphism developed independently in the *T. ferruginea* species group. This brings the number of known cases of independent development of sexual dimorphism in the Diopsidae to ten.

## Introduction

Five species and three genera of Diopsidae were known to occur in Sri Lanka (Feijen 1998). The genera *Diopsis* Linnaeus and *Sphyracephala* Say were both represented by one species, while *Teleopsis* Rondani counted three species. An overview will now be given of the rather limited literature on Diopsidae from Sri Lanka. The *Teleopsis* species in Sri Lanka belong to the *Teleopsis ferruginea* species group and this group will now be revised, expanding the group to five species. In the collections of NHMUK, two *Teleopsis* specimens were found which represent an undescribed species. These specimens will here be described as *Teleopsis neglecta* sp. nov. In the collections of NHMB, two male *Teleopsis* specimens were found which proved to be conspecific with *Teleopsis ferruginea* Röder of which the female holotype was fixed by monotypy. Feijen (1998) examined the teneral holotype and gave a redescription of *T. ferruginea* based on a series of 50 specimens. Comparison of the holotype and the two NHMB males with the series of 50 specimens showed that in fact two closely related species were involved. This issue will now be resolved by redescribing *T. ferruginea* based on its holotype and the two NHMB males, while as new species *Teleopsis sorora* sp. nov. will be described based on the series of 50 specimens and some additional specimens. Additional information will also be given for the two other *Teleopsis* species in Sri Lanka: *T. krombeini* Feijen and *T. maculata* Feijen. These species will be illustrated with photographs of the holotype and/or paratype.

In the collection of ZMUO, three specimens of *Cyrtodiopsis* Frey with Ceylon (Sri Lanka) labels were found. These belong to the *Cyrtodiopsis dalmanni* species group and would form a remarkable extension of the range of genus and species group. A key will be presented to the eight species now known to occur in Sri Lanka. The three species of the genera *Sphyracephala*, *Cyrtodiopsis* and *Diopsis* will be listed and illustrated. Allometric aspects with regard to the sexual dimorphism of the eye stalks in the *Teleopsis ferruginea* species group will be discussed. Allometric data will also be presented for the monomorphic *Diopsis* species.

## Material and methods

The description of *T. sorora* sp. nov. is based on a large series of pinned specimens. For the description of *T. neglecta* sp. nov. only two pinned specimens in rather poor condition were available, one specimen lacking the abdomen, while a male specimen lacked the head. Fortunately, five photographs of live specimens became available via www.iNaturalist.org. From the same source also photographs for *T. krombeini* and *T. sorora* sp. nov. were obtained. The redescription of *T. ferruginea* (Röder, 1893) is based on the rather teneral female holotype and two pinned male specimens. For the rate of dimorphism D, the difference between males and females in allometric slope for eye span on body length is used in the Diopsidae (Baker and Wilkinson 2001). Details on procedures for preparing genitalia slides, and procedures for taking measurements are given in Feijen et al. (2018a). For information on morphological terminology and onphotographic equipment used, the reader is referred to the same source. The following institutional codens and abbreviations are used:

**NHMUK**The Natural History Museum, London, United Kingdom,

**CNMS**National Museum, Colombo, Sri Lanka,

**MLUH**Wissenschaftsbereich Zoologie, Martin-Luther-Universität, Halle (Saale), Germany,

**NHMB**Naturhistorisches Museum, Basel, Switzerland,

**RMNH** Naturalis Biodiversity Center (formerly Rijksmuseum van Natuurlijke Historie), Leiden, The Netherlands,

**USNM** National Museum of Natural History (formerly United States National Museum), Washington D.C., United States of America,

**ZSM**Zoologische Staatssammlung des Bayerischen Staates, München, Germany.

**D** Rate of Dimorphism,

**SE** Standard Error.

## Overview of literature on Sri Lankan Diopsidae

The literature on Diopsidae from Sri Lanka is rather limited. The first paper is by Röder (1893) and describes *Diopsis ferruginea* from southern Sri Lanka (Ceylon meridionalis). Wulp (1896) listed *Diopsis ferruginea* in his catalogue of Diptera from South Asia. Likewise, Brunetti (1907) listed *Diopsis ferruginea* in his catalogue of Oriental Diopsidae. In 1922, Senior-White described *Teleopsis bipunctipennis* from “five males and seven females, all in good condition, and all taken at one sweep of the net on leaf of a plant growing in the water at edge of the Suduganga river”. Frey (1928) assumed that *Diopsis ferruginea* should eventually be placed in *Megalabops* Frey. Curran (1936) reported *Diopsis ferruginea* from Mergui, India, which is now in southern Myanmar. However, Shillito (1940) stated that this was a misidentification and that it concerned *Cyrtodiopsis currani* Shillito. Shillito furthermore considered that *Diopsis ferruginea* should be placed in *Megalabops* and *Teleopsis bipunctipennis* in *Pseudodiopsis* Hendel. Shillito (1971) maintained these allocations for the two Sri Lanka species. Descamps (1957) mentioned *Diopsis ferruginea* and *Teleopsis bipunctipennis* in his catalogue. Steyskal (1972) still listed *Teleopsis bipunctipennis* as such, while he placed *Diopsis ferruginea* in *Teleopsis*. In 1977, Steyskal in his catalogue of Oriental Diopsidae listed *Pseudodiopsis bipunctipennis* and *Teleopsis ferruginea* from Ceylon. Feijen (1989) transferred *Pseudodiopsis bipunctipennis* to *Sphyracephala*. Feijen (1998) listed five Diopsidae species for Sri Lanka. *Sphyracephala bipunctipennis* and a *Diopsis* of the *indica* species group were dealt with in a key to the diopsids of Sri Lanka and briefly discussed. *Teleopsis ferruginea* was redescribed, while as new species *Teleopsis krombeini* and *Teleopsis maculata* were described. Feijen (2011) placed these three *Teleopsis* in the *Teleopsis ferruginea* species group of Sri Lanka and described it as a distinct and aberrant species group in its genus. Feijen & Feijen (2019) indicated *T. ferruginea* as anendemic species of Sri Lanka, while the *T. ferruginea* species group was thought to form an isolated group in its genus. *Sphyracephala bipunctipennis* was reported from Tamil Nadu, India, so it no longer qualified as an endemic species of Sri Lanka.

## Taxonomy

**Family Diopsidae Billberg, 1820**

Diopsidae: Billberg 1820: 115 (as Natio Diopsides). Type genus: *Diopsis* Linnaeus, 1775: 5.

### Genus Teleopsis

Taxon classificationAnimaliaDipteraDiopsidae

Rondani, 1875

A56D510F-3DD2-5672-A879-742774C2664B

Figures 1–2 , 3–6 , 7–11 , 12–16 , 17–21 , 22, 23 , 24–27 , 28 , 29–32 , 33 , 34, 35 , 36–40 , 41–47 , 48–50 , 51 , 52–55 , 56–61 , 62 , 63

Teleopsis Rondani, 1875: 442; Feijen 1998: 49 (diagnosis, catalogue, discussion); Baker et al. 2001: 92 (cladogram, phylogenetic position within Diopsidae); Feijen 2011: 80 (discussion of taxonomic position); Feijen and Feijen 2011: 143 (biogeographic range). Type species: Diopsis sykesii Westwood, 1837 [= T. fulviventris Bigot, 1880 and T. onopyxus Séguy, 1949], by original designation.
Cyrtodiopsis Not Frey, 1928: 70; Shillito 1940: 156 (revision of Cyrtodiopsis); Feijen 1981: 480 (note on Cyrtodiopsis, disagreeing with the various synonymies of C. dalmanni proposed by Shillito 1940); Baker and Wilkinson 2001: 92 (Teleopsis paraphyletic and embedded within Cyrtodiopsis); Meier and Baker 2002: 332 (designation of synonymy); Liu et al 2009: 57 (maintaining Cyrtodiopsis); Feijen 2011: 80 (rejection of synonymy). Type species Diopsis dalmanni Wiedemann, 1830, by original designation.
Megalabops Not Frey, 1928: 70; Steyskal 1972: 11 (designation of synonymy); Feijen 1989: 62 (supporting synonymy); Baker et al., 2001, (supporting synonymy based on molecular analyses); Feijen 2011: 80 (re-instating Megalabops); Feijen and Feijen 2019: 48 (discussion of taxonomic position). Type species, Diopsis quadriguttata Walker, 1857, by original designation.

### Teleopsis ferruginea

Taxon classificationAnimaliaDipteraDiopsidae

(Röder, 1893)

AB6DF533-646D-59A4-87A1-2ACEF53209BF

Figures 1–2 , 3–6 , 7 , 12–16 , 17 , 51 , 62 , 63

Diopsis ferruginea Röder, 1893: 235; Wulp 1896: 171; Brunetti 1907: 165; Descamps 1957: 18.
Megalabops ferruginea ? (Röder): Frey 1928: 70.
Megalabops ferruginea (Röder): Shillito 1940: 157.
Teleopsis ferruginea (Röder): Steyskal 1972: 11, 1977: 34; Feijen 1998: 55 (record of holotype only, redescription based on “Further material” now referred to Teleopsis sorora sp. nov.).
Teleopsis ferruginea Not: Curran, 1936: 2 (= Cyrtodiopsis currani Shillito, 1940).
Teleopsis ferruginea Not: Kotrba et al. 2013: 190 (= Teleopsis sorora sp. nov.).

#### Type material.

***Holotype***, ♀, [Sri Lanka], Ceylon meridionalis [South Sri Lanka], v.1889, H. Fruhstorfer (MLUH).

#### Material studied.

Holotype ♀; 2 ♂, Uva, Lunugala, [7°2'26"N, 81°12'06"E, ~760 m], 25.ix.[19]53, F. Keiser (NHMB).

#### Diagnosis.

*Teleopsis ferruginea* can be recognised by its size, slender habitus, bareness, wing pattern (apical infuscation, three crossbands, broad preapical crossband, irregular central crossband with darker patches along veins, irregular narrow basal crossband, two small pale spots between basal and central crossbands, two distinct clear spots between central and preapical crossbands), wing mostly covered by microtrichia except for bare spots on basal third, small, setula-like inner vertical seta 0.5× the stalk diameter, outer vertical seta 1.4× stalk diameter, tiny base of inner vertical seta, no facial teeth, pollinose collar, reddish brown, thinly pollinose scutum and scutellum, ratio scutellar spine/scutellum ~ 2.8, moderately incrassate front femora with around 44–60 (♂) tubercles, large glossy spot laterally on terga 1 and 2, abdomen brown with dark parts of terga 3–5 forming a black circle, pair of pollinose spots on tergum 3, left male spiracle 7 in lateral slit of synsternum, right spiracle 7 in synsternum, articulate surstyli very small, apically rounded (as long as wide in lateral view), surstyli without microtrichia, large male cerci apically pointed (ratio length/width 1.6), anterior arm of phallapodeme quite straight, only slightly curving downward anteriorly, ratio eye span/body length 0.81 in ♀, 0.85 and 1.01 in ♂, and assumed sexual dimorphism with regard to eye span of ~ 0.8. *Teleopsis ferruginea* can be considered the sister species of *T. sorora* sp. nov. and gives its name to the *T. ferruginea* species group.

#### Description.

**Figures 1, 2.**
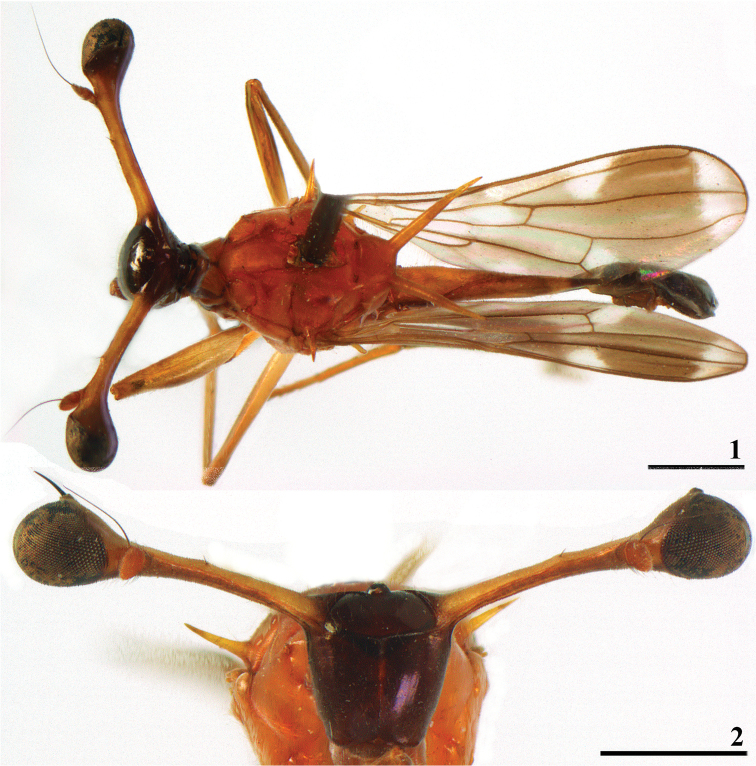
*Teleopsis ferruginea*, ♀, holotype **1** habitus, dorsal view **2** head, anterior view. Scale bars: 1 mm.

***Measurements*.** Body length holotype ♀ 6.3 mm (estimate, specimen is teneral, see Fig. 1), 2 ♂ respectively 4.8 and 6.8 mm; eye span holotype 5.1 mm, 2 ♂ 5.6 and 6.8 mm; wing length holotype 4.8 mm, 2 ♂ 4.0 and 4.8 mm; length of scutellar spine holotype 1.24 mm, 2 ♂ 0.96 and 1.25 mm.

**Figures 3–6.**
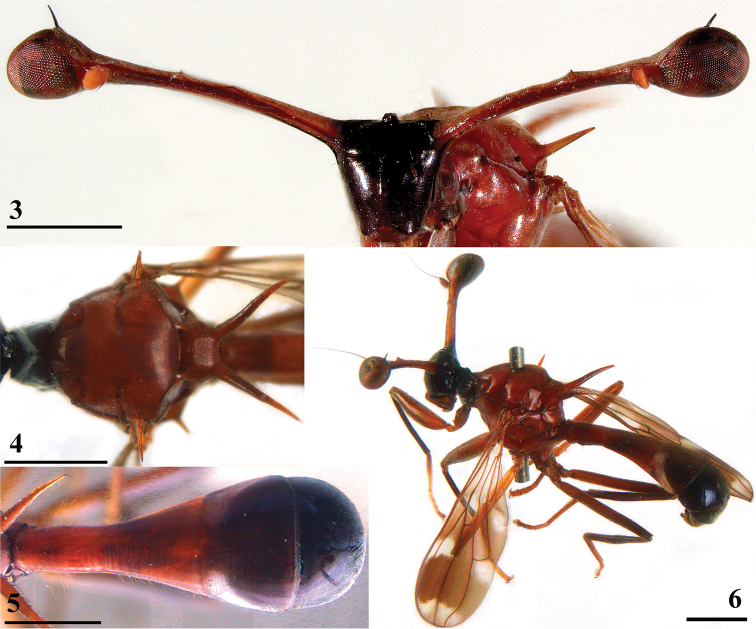
*Teleopsis ferruginea*, ♂, Lunugala **3** head, anterior view **4** thorax, dorsal view **5** abdomen, dorsal view **6** habitus, lateral view (**3, 5** larger ♂, **4, 6** smaller ♂). Scale bars: 1 mm.

***Head*.** Central part glossy dark brown, almost black (Figs 2, 3), face laterally with some very fine pollinosity; frons (Figs 2, 3) very smooth, surrounded by simple, semi-circular ridge; arcuate groove thin and concolourous; face very smooth, no facial teeth, lateroventral corners rounded, almost bare, a few tiny pale setulae; eye span in holotype ♀ small (19% shorter than body length) and small to medium-sized in the two males (15% shorter than body length in the small male and 1% longer than body length in the large male); probably moderate rate of dimorphism in eye span, comparison of the three data points with the graph for *T. sorora* sp. nov. (see Fig. 51) indicates a D of around 0.8; stalks brown, broad apical parts blackish, dorsal part of stalks pollinose; inner vertical seta small and setula-like in the ♀, just more than 0.5× the diameter of the stalk, in the two ♂ the inner vertical seta is likely to be broken off, base of inner vertical seta small, just more than 0.1× the stalk diameter; outer vertical seta 1.4× stalk diameter.

***Thorax*.** Collar brown pollinose, but anteriorly and laterally more blackish brown; scutum, scutellum and scutellar spines reddish brown (ferruginous), thinly pollinose (Figs 1, 4); pleura glossy brown, only some pollinosity on anterior and posterior margins; supra-alar spines (Figs 1, 4) glossy brown, almost 3× as long as pleurotergal spines, dorsolaterally directed; scutellar spines almost straight, diverging under an angle of about 75° (Figs 1, 4), ratio scutellar spine/scutellum in holotype ♀ 2.76, in the small ♂ 2.67 and in the large ♂ 2.89, ratio scutellar spine/body length in holotype 0.20, in small ♂ 0.17 and in large ♂ 0.19; pleurotergal spines pollinose, medium-sized and blunt, posterolaterally directed; apical seta small (13% of length of scutellar spine in holotype, partly broken off in ♂); tiny white setulae on thorax, scutellar spines without warts, only with tiny setulae.

**Figures 7–11.**
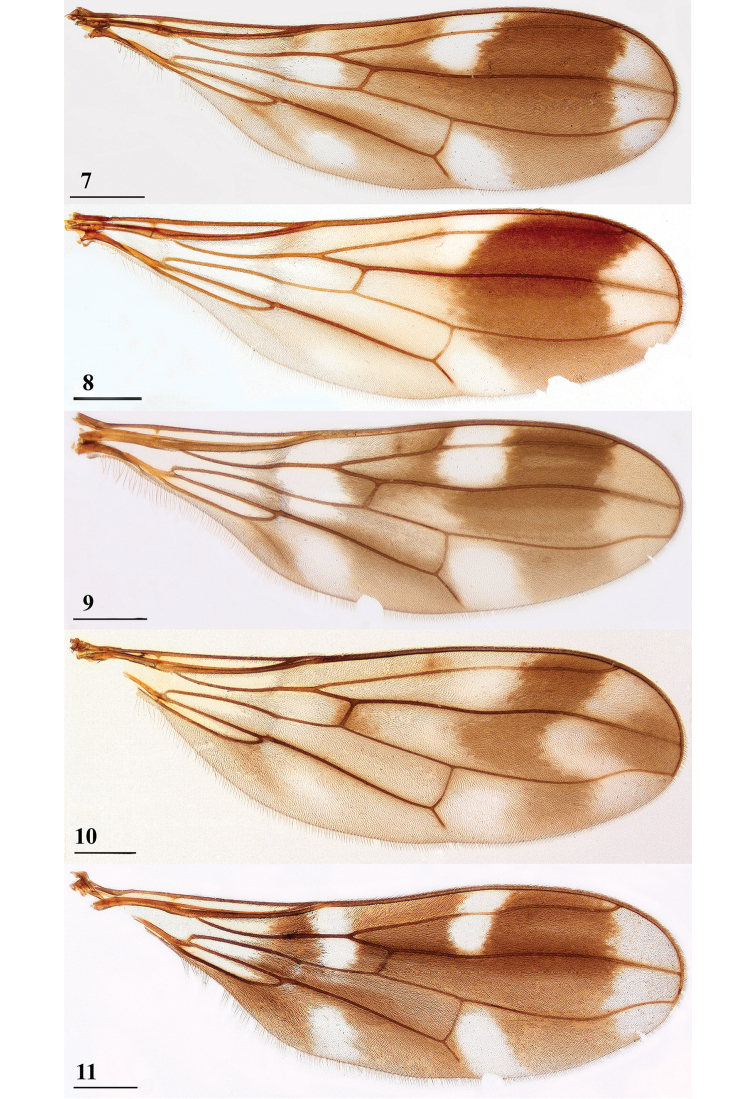
Dorsal view of *Teleopsis* wings **7***T. ferruginea*, ♂, Lunugala **8***T. sorora* sp. nov., ♂, paratype, Roseneath **9***T. krombeini*, ♂, paratype, Thawalamtenne **10***T. maculata*, ♂, paratype, Hakgala **11***T. neglecta* sp. nov., unknown sex, paratype, Pundaluoya. Scale bars: 0.5 mm.

***Wing*.** Irrorated with three crossbands (Figs 1, 7); apex (apical 6% of wing) distinctly infuscated, infuscated area linked to preapical band along veins and wing edge; preapical band broad, almost uniformly dark, posteriorly slightly paler, broadly linked tocentral crossband in cell r4+5, slightly extending into cells r2+3 and m; two clear spots in between the central and preapical bands, one in cells r1 and r2+3, and one basally in cell m1; broad, but irregular central crossband including crossveins r-m and dm-m, darker in cell r1 and around veins R4+5 and M4; irregular basal band narrow, darker in cell r1 and around vein M4, several connections to central band, giving two pale spots in cell br and cell m4, a vague dark stripe running from cell cua to the pale spot in cell m4; cell r4+5 narrower basally and apically; vein M4 from crossvein dm-m onward turning downward and reaching till more than three-quarters of the distance to the wing edge; glabrous basal areas including basal half of cell c, tiny basal spot in cell r1, basal half of cell br, basal quarter of cell bm+dm except for posterior edge, and posterior half of cell cua.

***Legs***. Front leg with brown coxa, trochanter and femur, coxa glossy on outer side and pollinose on inner side, femur pollinose with vague darker spot on outer side, tibia and basal half of metatarsus blackish brown, remainder of tarsus very pale brown; mid leg and hind leg brown, hind femur and hind tibia dark brown; femur 1 (Figs 1, 6) moderately incrassate (ratio of length/width in ♀ 4.7 and in both ♂ 4.5), tubercles on distal three-quarters of ventral side, inner row in ♀ with 23 tubercles and in ♂ with 28.5 ± SE 2.6tubercles (range 24–34, *N* = 4), outer row in ♀ with 20 tubercles (range 19–21) and in ♂ with 23.0 ± 1.7 tubercles (range 20–26, *N* = 4), in both rows a few double tubercles.

**Figures 12–16.**
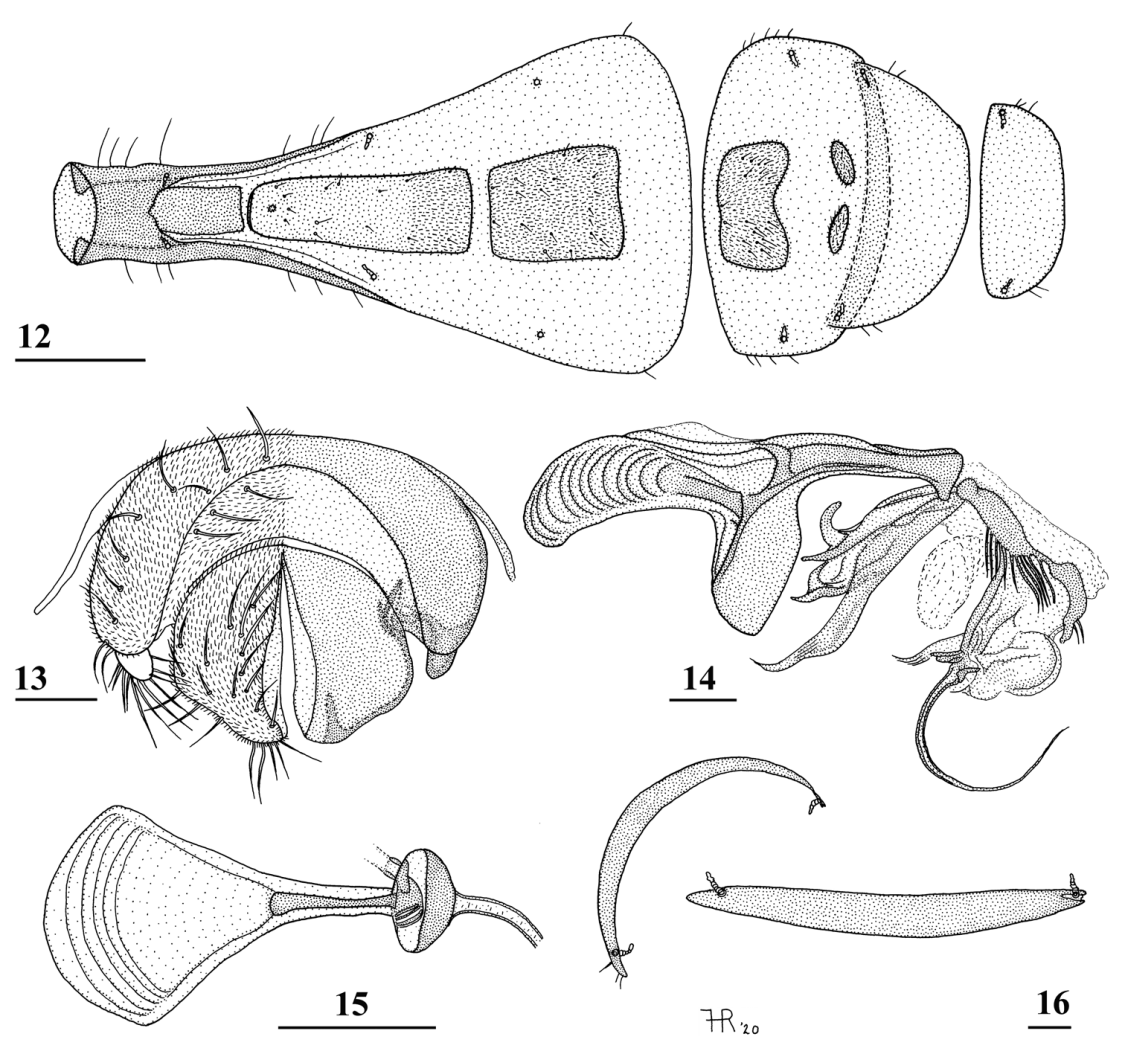
*Teleopsis ferruginea* ♂, Lunugala **12** six basal abdominal segments, ventral view (note absence of sternum 6) **13** epandrium with surstyli and cerci, posterior view **14** phallapodeme and aedeagus, lateral view **15** ejaculatory apodeme and sac **16** synsternum 7+8, natural, curved state in dorso-anterior view and stretched state in ventral view. Scale bars: 0.5 mm (**12**); 0.1 mm (**13–16**).

***Preabdomen*.** Terga 1 and 2 and base of tergum 3 brown, remainder of tergum 3, terga 4, 5 and 6 blackish, the dark parts of terga 3–5 forming a black circle (Figs 5, 6); terga thinly pollinose, a glossy lateral spot on tergum 1 and basal half of tergum 2, anterolaterally on tergum 3 a pair of densely pollinose spots; seam between terga 2 and 3 not very distinct; sternum 1 and intersclerite brown, sternum 1 more glossy basally, other sterna pale yellowish brown, pollinose; basal half of sternum 1 seamlessly fused to tergum 1; spiracle 1 in tergum; intersclerite posteriorly connected to sternum 2 (Fig. 12), sternum 2 narrow and long, slightly broadening posteriorly; rectangular sternum 3 slightly broader than sternum 2, sternum 4 again slightly broader and more or less rectangular (Fig. 12).

***Female postabdomen*.** Given the teneral and damaged state (Fig. 1) of the abdomen of the holotype ♀, it was decided not to dissect the abdomen.

**Figures 17–21.**
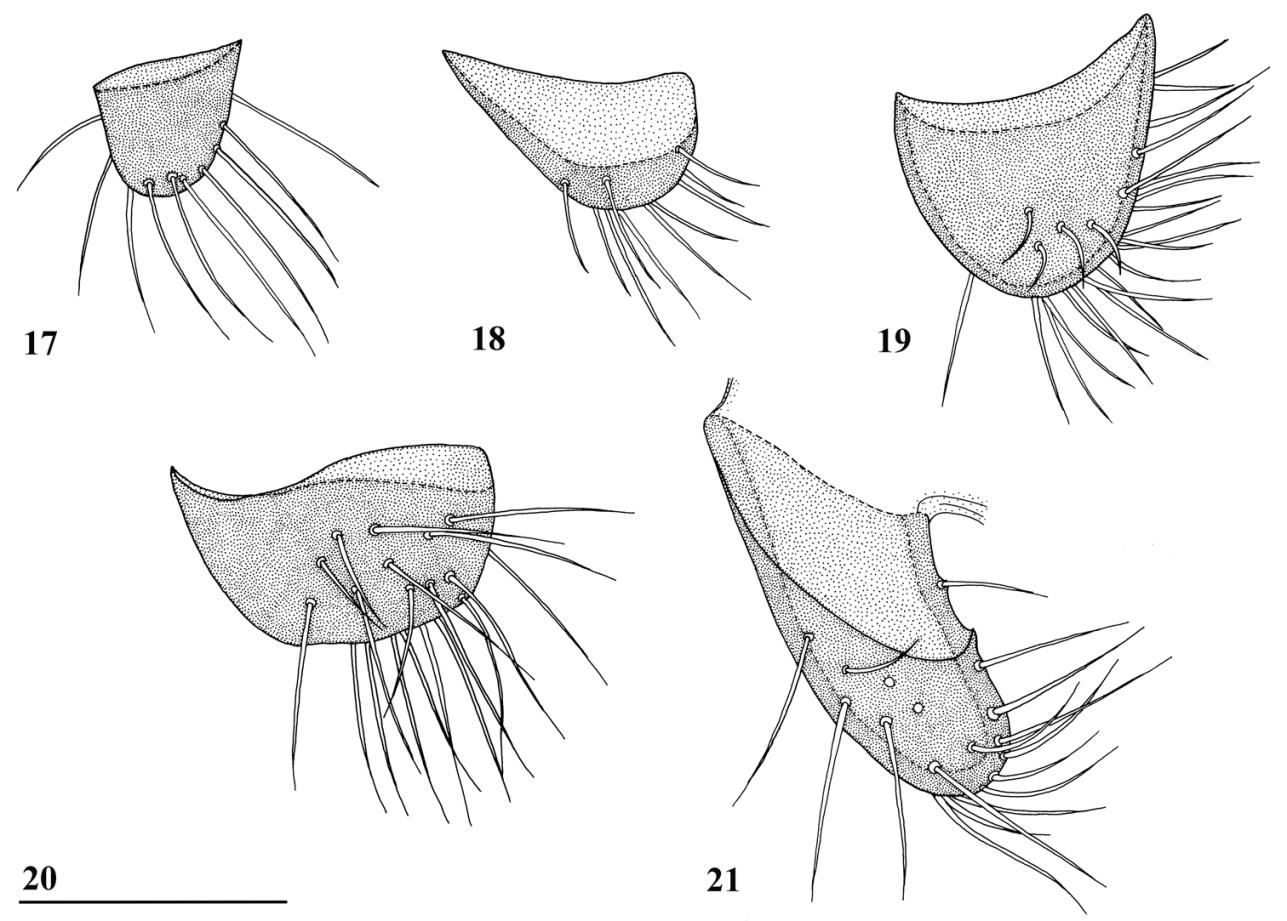
Lateral view of *Teleopsis* surstyli **17***T. ferruginea*, Lunugala **18***T. sorora* sp. nov., paratype, Udawattakele **19***T. krombeini*, paratype, Thawalamtenne **20***T. maculata*, paratype, Hakgala **21***T. neglecta* sp. nov., holotype, Pundaluoya. Scale bar: 0.1 mm (all drawn to the same scale).

***Male postabdomen*.** Strongly deflexed, sternum 5 represented by two very small, strongly sclerotised sclerites (Fig. 12), sternum 6 hardly discernible: synsternum 7+8 (Fig. 16, on the left the natural, curved position and at right the flattened shape); left spiracle 7 in vague lateral slit of synsternum, right spiracle 7 in synsternum at anterior edge; epandrium (Fig. 13) rounded, covered with microtrichia and about 11 pairs of setulae, anterior section largely separated; surstyli articulate, very small, apically rounded, in lateral view symmetrical and rounded, ratio length/width 1.0 (Fig. 17), no microtrichia, at apical edge 10 setulae, setulae about 1.5 times as long as surstylus; surstyli just connected to lateral side of cerci, not interconnected via processus longi; cerci large, apically pointed towards the meson (Fig. 13), ratio length/width 1.6, widest subapically, covered with microtrichia and about 20 setulae; phallapodeme (Fig. 14) quite straight, anterior arm slightly curving downward anteriorly, anteriorly rounded, anterior arm marginally longer than posterior arm, posterior arm bifurcated, vane not very broad; aedeagus a complicated open structure of sclerites and membranes (Fig. 14), rather long “male genital process” sticking out from apex (for terminology see Kotrba et al. 2013); ejaculatory apodeme wedge-shaped (Fig. 15).

#### Distribution.

*Teleopsis ferruginea* occurs in the Uva Province in south-eastern Sri Lanka. The holotype originates from southern Sri Lanka.

### Teleopsis krombeini

Taxon classificationAnimaliaDipteraDiopsidae

Feijen, 1998

030C17D7-B5D0-5A2D-A159-898E859B882C

Figures 9 , 19 , 22–23 , 24 , 28 , 29–30 , 62 , 63

Teleopsis krombeini Feijen, 1998: 57.

#### Type material.

***Holotype***, ♂, Sri Lanka, Kan[dy] Dist[rict], Thawalamtenne, 2200 ft, 4.ix.1980, K.V. Krombein et al. (USNM). ***Paratypes***: 4 ♀, 6 ♂ and 2 ?sex, same data as holotype (USNM, RMNH); 1 ♀, Kitulgala, Bandarakele Jungle, Keg[alle] Dist., 17–18.iii.1979, K. V. Krombein et al. (USNM); 3 ♀ Kandy, 28.v.1892, Lt Col. Yerbury (NHMUK); 2 ♂, [Kandy District], Haragam[a], 24.v.1892, Lt Col. Yerbury (NHMUK); 1 ♂ (NHMUK genitalia slide), Kandy, 24.v.1892, Lt Col. Yerbury; 1 ♀ (NHMUK genitalia slide), Haragam[a], 1.vi.1892, Lt Col. Yerbury.

#### Additional material.

Photographs (www.inaturalist.org/observations/29425824) by Amila Prasanna Sumanapala taken at Central Province, Matale District, Rattota, 7°31'05"N, 80°43'52"E, 1155 m, 27.ii.2019. Although these pictures were sufficient for identification, they were not sharp enough to be reproduced here.

#### Notes.

**Figures 22, 23.**
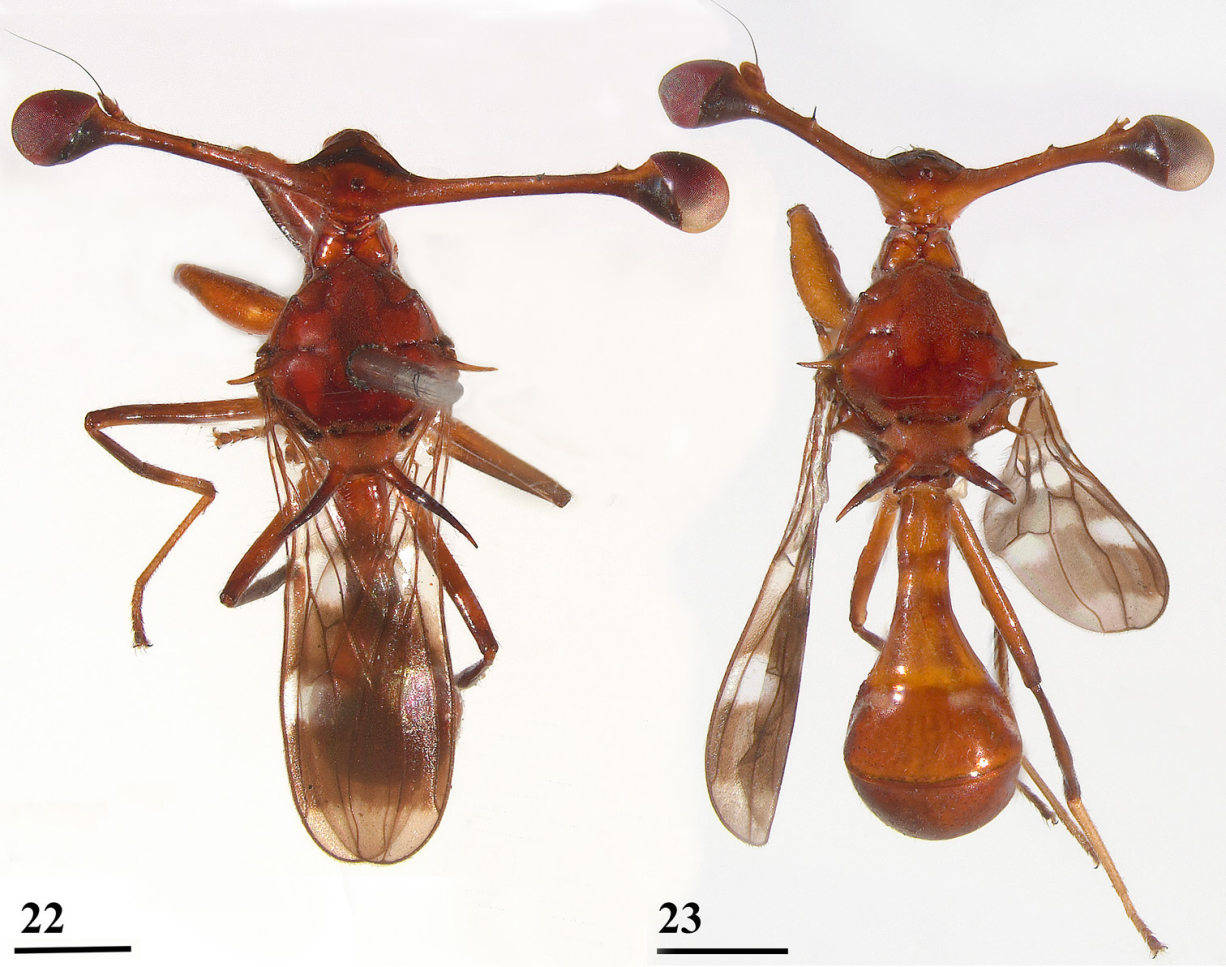
Dorsal view of *Teleopsis krombeini***22** ♂, holotype, Thawalamtenne **23** ♀, paratype, Kitugala. Scale bars: 1 mm.

**Figures 24–27.**
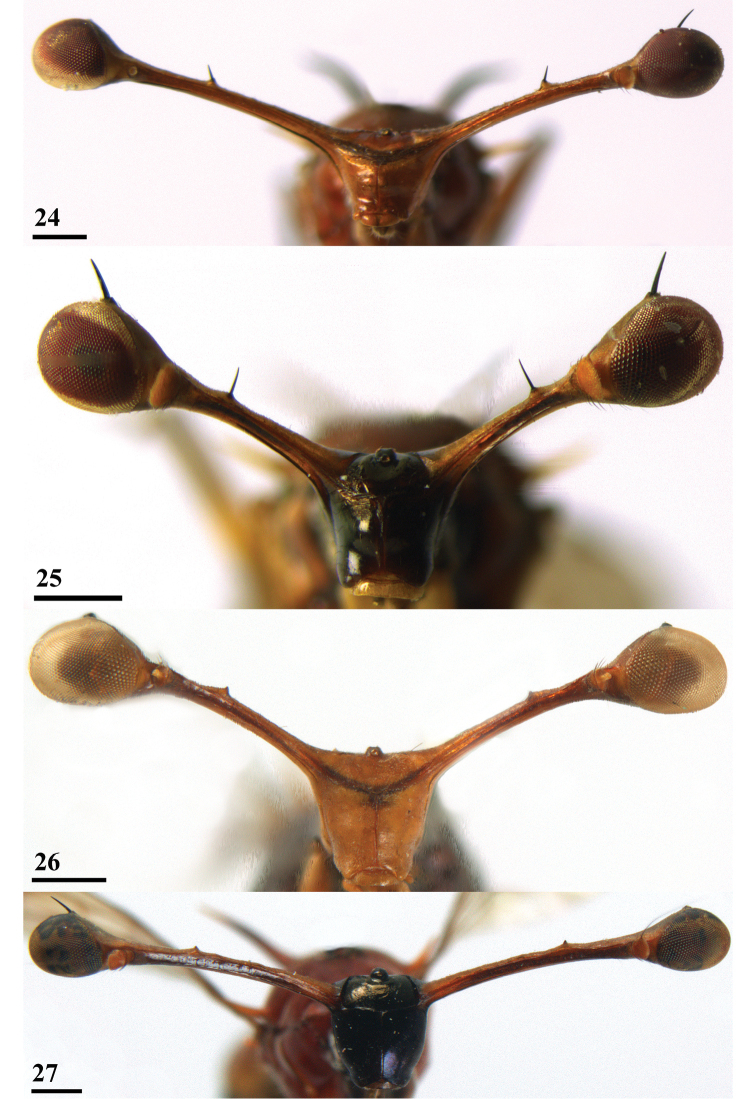
Anterior view of *Teleopsis* heads **24***T. krombeini*, ♂, holotype, Thawalamtenne **25***T. maculata*, ♂, paratype, Hakgala **26***T. neglecta* sp. nov., unknown sex, paratype, Pundaluoya **27***T. sorora* sp. nov., ♂, paratype, Roseneath. Scale bars: 0.5 mm.

Holotype and paratypes from USNM and RMNH were re-examined. Holotype ♂ and a paratype ♀ were photographed (Figs 9, 22–24). The re-examinedflies were measured. Below, only data additional to the description given in Feijen (1998) are presented.

***Measurements***. Body length ♀ 5.9 mm ± SE 0.2 (range 5.4–6.5, *N* = 5), ♂ 5.9 mm ± 0.2 (range 5.0–6.4, *N* = 7); eye span ♀ 4.6 mm ± 0.1 (range 4.2–5.0, *N* = 5), ♂ 5.7 mm ± 0.4 (range 4.2–6.8, *N* = 7); wing length ♀ 4.3 mm ± 0.1 (range 4.0–4.7, *N* = 5), ♂ 4.4 mm ± 0.1 (range 3.8–4.7, *N* = 7); length of scutellar spine ♀ 1.14 ± 0.05 (range 1.04–1.30, *N* = 5), ♂ 1.11 mm ± 0.05 (range 0.89–1.25, *N* = 7).

**Figure 28.**
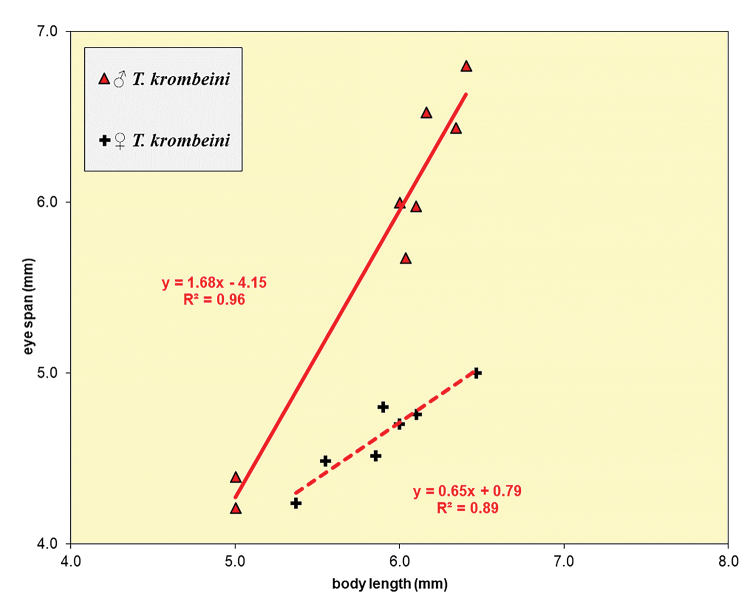
*Teleopsis krombeini*, eye span plotted against body length.

***Head***. Eye span (Figs 22–24) small in female (78.4 ± 0.7% of body length) and medium-sized in male (96.8 ± 3.3% of body length); a dimorphic species with a moderate rate of dimorphism D = 1.03 (Fig. 28); inner vertical seta small, equal in size to stalk diameter, usually not broken off; base of inner vertical seta small, almost 0.5× the stalk diameter; outer vertical seta medium-sized, about 1.7× the stalk diameter, spinous.

***Thorax.*** Ratio scutellar spine/scutellum in ♀ 3.16 ± 0.06 (*N* = 5) and in ♂ 3.13 ± 0.05 (*N* = 7), ratio scutellar spine/body length in ♀ and ♂ 0.19 ± 0.00 (*N* = 5, resp. 7); apical seta small, one-fifth of length of scutellar spine (lacking in most specimens).

***Wing***. See Fig. 9.

**Figures 29–32.**
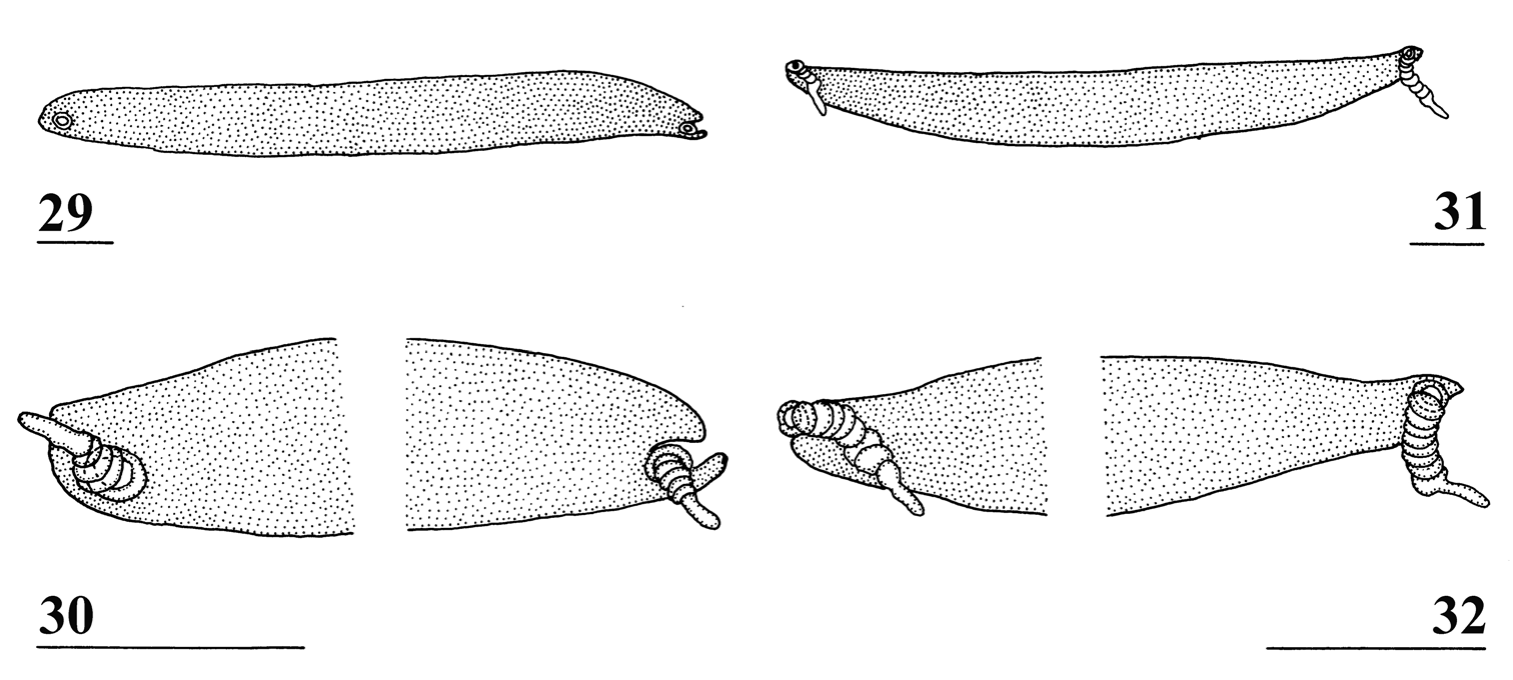
Ventral view of *Teleopsis* male synsternum 7+8 **29***T. krombeini*, paratype, Thawalamtenne **30** same, details of lateral sections **31***T. maculata*, paratype, Hakgala **32** same, details of lateral sections. Scale bars: 0.1 mm.

***Male postabdomen***. Sternum 6 indiscernible; synsternum 7+8 a short transverse sclerite with parallel anterior and posterior edges (Fig. 29), right spiracle 7 in sclerite, left spiracle 7 in slit in lateral tip of synsternum (Fig. 30) [for the remark by Feijen (1998) “left spiracle 7 in sternum 7+8, right spiracle 8 in lateral slit of sternum 7+8 (fig. 20)” left and right have to be reversed as is also clear from the figure]; surstyli articulate, small (but relatively large, as compared to *T. ferruginea* and *T. sorora* sp. nov.), apically rounded, in lateral view symmetrical and rounded, ratio length/width 0.9 (Fig. 19), no microtrichia, at apical and posterior edges with about 25 setulae, setulae distinctly shorter than length of surstylus.

#### Distribution.

*Teleopsis krombeini* is now known from Kandy District and Matale District, Central Province and Kegalle District, Sabaragamuwa Province.

### Teleopsis maculata

Taxon classificationAnimaliaDipteraDiopsidae

Feijen, 1998

6827D341-8C50-5656-9A01-6E6196B496EA

Figures 10 , 20 , 25 , 31–32 , 33 , 62 , 63

Teleopsis maculata Feijen, 1998: 61.

#### Type material.

***Holotype***, ♂, Sri Lanka, Nuwara Eliya, 14.vii.1892, Lt Col. Yerbury (NHMUK). ***Paratypes***: 1 ♂, Hakgala Natural Reserve, N.E. Dist., Sri Lanka, 6–7.ii.1979, K. V. Krombein, P. B. Karunaratne, T. Wijesinhe, S. Siriwardane, T. Gunawardane (USNM); 1 ?sex (no head and abdomen), Punda luoya [Pundaloya], Sri Lanka, E. E. Green (NHMUK).

#### Notes.

**Figure 33.**
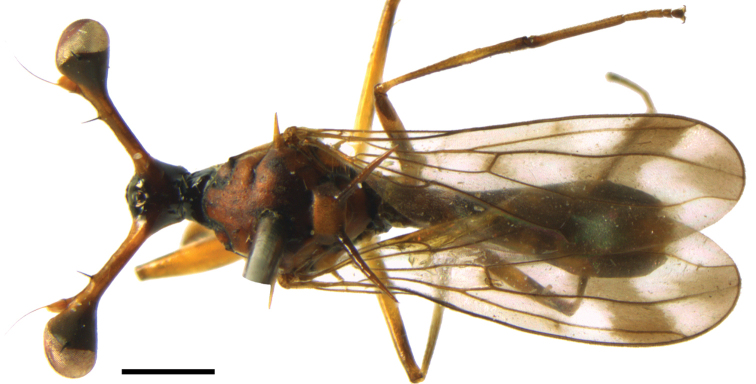
*Teleopsis maculata*, ♂ paratype, Hakgala, dorsal view. Scale bar: 1 mm.

The USNM paratype was re-examined and photographed (Figs 10, 25, 33). Below, only data additional to the description given in Feijen (1998) are presented.

#### Diagnosis.

*Teleopsis maculata* forms part of the *T. ferruginea* species group. For the position of this monomorphic species within this group can be referred to the remarks made under *T. neglecta* sp. nov.

#### Description.

***Head***. Eye span (Figs 25, 33) very small in male (62.4 ± 1.7% of body length); although no females are available, the very small eye spans in the two available males form a clear indication that this is a monomorphic species; when thetwo data points for ratio eye span/body length in *T. maculata* (Figs 62, 63) are compared with the data points for the dimorphic *T. ferruginea*, *T. krombeini* and *T. sorora* sp. nov., these two points are in slope similar to the females of the three dimorphic species; inner vertical seta medium-sized, about 1.5× the stalk diameter; base of inner vertical seta small, less than 0.5× the stalk diameter; outer vertical seta medium-sized, about 2.0× the stalk diameter, spinous (Figs 25, 33).

***Thorax.*** Ratio scutellar spine/scutellum in paratype ♂ 3.00, ratio scutellar spine/body length in holotype 0.20 and in paratype ♂ 0.19; ratio apical seta/scutellar spine in holotype 0.27 and in paratype ♂ 0.29.

***Wing***. See Fig. 10.

***Male postabdomen***. Sternum 6 indiscernible; synsternum 7+8 (Fig. 31) a symmetrical, short, transverse sclerite, slightly curved and tapering laterally; spiracles 7 in synsternum near the lateral tips (Fig. 32); surstyli articulate, small, apically somewhat rounded, in lateral view distinctly wider than long, ratio length/width 0.6 (Fig. 20), no microtrichia, on large, distoposterior section about 19 setulae, most setulae slightly longer than length of surstylus.

#### Distribution.

*Teleopsis maculata* is known from the Central Province (Nuwara Eliya district). As the Hakgala Reserve is partly located in Uva Province, it probably also occurs there.

### Teleopsis neglecta sp. nov.

Taxon classificationAnimaliaDipteraDiopsidae

0798B9CB-9C20-527C-B092-8903D7416E1C

http://zoobank.org/9772DF96-ACAD-4DC9-BC70-F7CF807939D4

Figures 11 , 21 , 26 , 34–35 , 36–40 , 41–47 , 62 , 63

#### Type material.

***Holotype***, ♂, Ceylon [Sri Lanka, Central Province], Pundaluoya, [7°0'47"N, 80°39'48"E, ~1060 m], 90–115 [undated, but 90–115 probably indicates1890], E. E. Green (NHMUK), [head lacking]. ***Paratype***: 1 ? sex, same data as holotype [abdomen lacking].

#### Additional material.

**Figures 34, 35.**
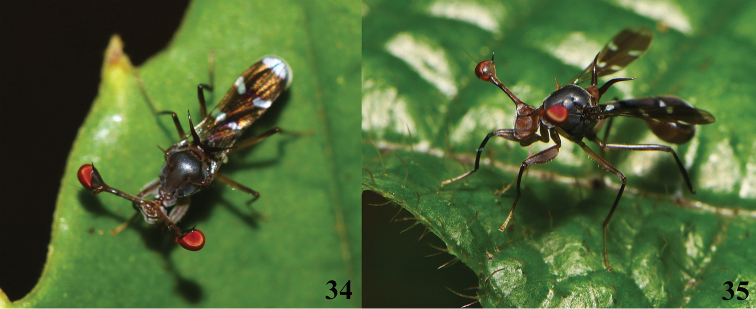
*Teleopsis neglecta* sp. nov., live photographs **34** by Amila Prasanna Sumanapala, Kalawana (www.inaturalist.org/observations/29425825) **35** by “Baeru”, Sinharaja Rainforest (www.inaturalist.org/observations/45907083).

Photographs: 1 ♂?, Sabaragamuwa province, Ratnapura, Kalawana, 6°25'12"N, 80°25'05"E, 510 m, 6.xi.2018 by Amila Prasanna Sumanapala (Fig. 34) (www.inaturalist.org/observations/29425825); 1 ♂, Southern Province, Matara, Kotapola, Sinharaja Forest Reserve, 6°21'47"N, 80°29'19"E, 420 m, 28.xi.2019 by “Baeru” (www.inaturalist.org/observations/45756128); 1?sex, Southern Province, Matara District, Kotapola, Sinharaja Forest Reserve, 6°21'45"N, 80°29'13"E, 350 m, 28.xi.2019 by “Baeru” (www.inaturalist.org/observations/45756204); 1 ♀, Sabaragamuwa Province, Ratnapura, Kalawana, Sinharaja Rainforest, 6°24'01"N, 80°29'58"E, 860 m, by “Baeru” (Fig. 35) (www.inaturalist.org/observations/45907083).

#### Diagnosis.

*Teleopsis neglecta* sp. nov. can be recognised by its slender habitus, bareness, wing pattern (apical 10% vaguely infuscated, three distinct crossbands strongly interconnected giving four distinct pale spots, basal anterior spot not extending into cell bm+dm), wing mostly covered by microtrichia except for most of basal quarter and anterior spots, inner vertical seta and outer vertical seta spinous, tiny base of inner vertical seta, no facial teeth, dorsally glossy collar, reddish brown, thinly pollinose scutum and scutellum, ratio scutellar spine/scutellum ~ 3.1, incrassate front femora with around 54 (♂) tubercles, abdomen dark, large glossy spot laterally on terga 1 and 2, pair of pollinose spots on tergum 3, tergum 4 glossy, tergum 5 densely pollinose, male spiracles 7 symmetrically in synsternum, surstyli articulate, slender, apically rounded, ratio length/width in lateral view 2.5, surstyli without microtrichia, broad male cerci, ratio eye span/body length ~0.60–0.70 in ♂, and assumed sexual monomorphism with regard to eye span.

*Teleopsis neglecta* sp. nov. forms part of the *T. ferruginea* species group. Like *T. maculata*, it is more distant from the dimorphic species in this group (*T. ferruginea*, *T. krombeini* and *T. sorora* sp. nov.), also given the differences in the male synsternum. However, for an understanding of the phylogenetic relationship between *T. neglecta* sp. nov. and *T. maculata* more information has to become available (morphology of female genitalia, molecular analyses and wing geometric morphometrics analyses).

#### Description.

***Measurements***. Body length holotype ♂ 6.9 mm (estimate: collar - abdominal apex 6.3 mm, head length assumed 0.6 mm); eye span paratype 4.8 mm; wing length holotype 4.1 mm and paratype 4.8 mm; length of scutellar spine holotype 1.08 mm and paratype 1.18 mm.

**Figures 36–40.**
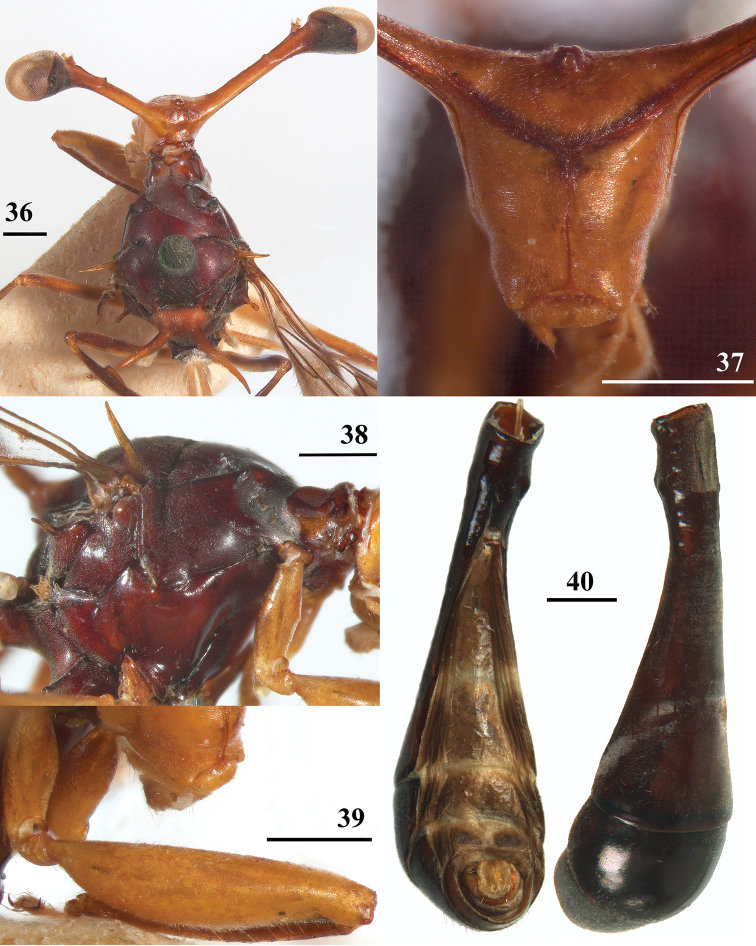
*Teleopsis neglecta* sp. nov. **36–39** unknown sex, paratype, Pundaluoya **40** ♂, holotype, Pundaluoya **36** head and thorax, dorsal view **37** central head, anterior view **38** thorax, lateral view **39** front femur, lateral view **40** abdomen, ventral (left) and dorsolateral (right) view. Scale bars: 0.5 mm.

***Head*.** Central part yellowish brown (Figs 34–37), thinly pollinose; frons (Fig. 37), smooth, with a shallow dimple in front of ocellar tubercle; arcuate groove dark brown; face smooth, slightly bulging centrally, no facial teeth, lateroventral corners rounded, bare, no setulae; eye span probably very small, in holotype estimated as ~60% of the body length (based on comparison of measurements of holotype and paratype), from the photograph of a live specimen an estimate of ~70% of body length is made; the rate of dimorphism in eye span cannot be estimated from the few data available, but it seems quite certain that *T. neglecta* sp. nov. is a monomorphic species; although no females are available, the very small eye span in the holotype forms a clear indication that this is a monomorphic species; when the data point for ratio eye span/body length in *T. maculata* (Figs 62, 63) is compared with the data points for the dimorphic *T. ferruginea*, *T. krombeini*, and *T. sorora* sp. nov., this point is similar to those for the females of the three dimorphic species [measurements are, like for *T. maculata* falling in the range for the monomorphic genus *Megalabops*]; stalks brown, broad apical parts blackish, thinly pollinose; inner vertical seta tiny, 0.1× the diameter of the eye stalk (Fig. 36), base of inner vertical seta small, 0.2× the stalk diameter; outer vertical seta broken off, but spinous (distinct in live photograph, Figs 34, 35).

***Thorax***. Collar dorsally glossy brown, but posteriorly and laterally pollinose; scutum reddish brown pollinose (darker in live photographs, Figs 34, 35), scutellum and scutellar spines yellowish brown (darker in live flies), thinly pollinose (Figs 36, 38); pleura reddish brown pollinose, anepisternum, anepimeron, katepisternum, and meron glossy reddish brown; supra-alar spines (Figs 36, 38) glossy brown, almost 2½x as long as pleurotergal spines, dorsolaterally directed; scutellar spines curving upward and outward, diverging under an angle of 100° (Figs 34–36), ratio scutellar spine/scutellum in holotype 3.21 and in paratype 3.06, ratio scutellar spine/body length in holotype ~ 0.16; pleurotergal spines pollinose, medium-sized and blunt, posterolaterally directed; apical seta broken off in holotype and paratype, but on photograph small, ~ 20% of length of scutellar spine; no setulae on thorax, a few tiny setulae on scutellar spines, no warts.

***Wing***. Irrorated with three distinct crossbands (Figs 11, 34, 35); apical 10% of wing uniformly vaguely infuscated; preapical band broad and dark, posterior half slightly paler, broadly linked to central crossband in cell r4+5, slightly extending into cells r2+3 and m; two clear spots in between the central and preapical bands, one in cells r1 and r2+3, and one basally in cell m1; central crossband dark and almost as wideas preapical band, including crossveins r-m and dm-m, darker veins R4+5 and M4, slightly less dark on posterior half, preapical and central crossband together forming a solid H-configuration; basal band dark and half the width of the other bands, darker around vein M4 and posteriorly of cell cua, a strong connection to central band incell bm+ dm and around vein M4, giving two pale spots, one in cells r1 and br and the other centrally in cell m4; cell r4+5 narrower basally and apically; vein M4 from crossvein dm-m onward turning slightly downward and reaching till just more than half the distance to the wing edge; glabrous basal areas including most of cell c except for apex, posterior basal sixth of cell r1, basal half of cell br, basal fifth of cell bm+dm except for posterior edge, and most of cell cua except for apex and anterior margin; anterior spots also almost bare.

***Legs***. Front leg yellowish brown pollinose (darker in live flies), femur with very vague brown stripe on inner side, tibia darker brown; mid leg brown, femur with dark brown stripes on distal half; hind leg brown, femur with dark brown stripe on whole length, hind tibia darker brown; femur 1 (Fig. 39) incrassate in paratype, ratio of length/width 3.9, tubercles on distal three-quarters of ventral side, inner row in paratype with 28 tubercles (*N* = 1), outer row with 26 tubercles.

**Figures 41–47.**
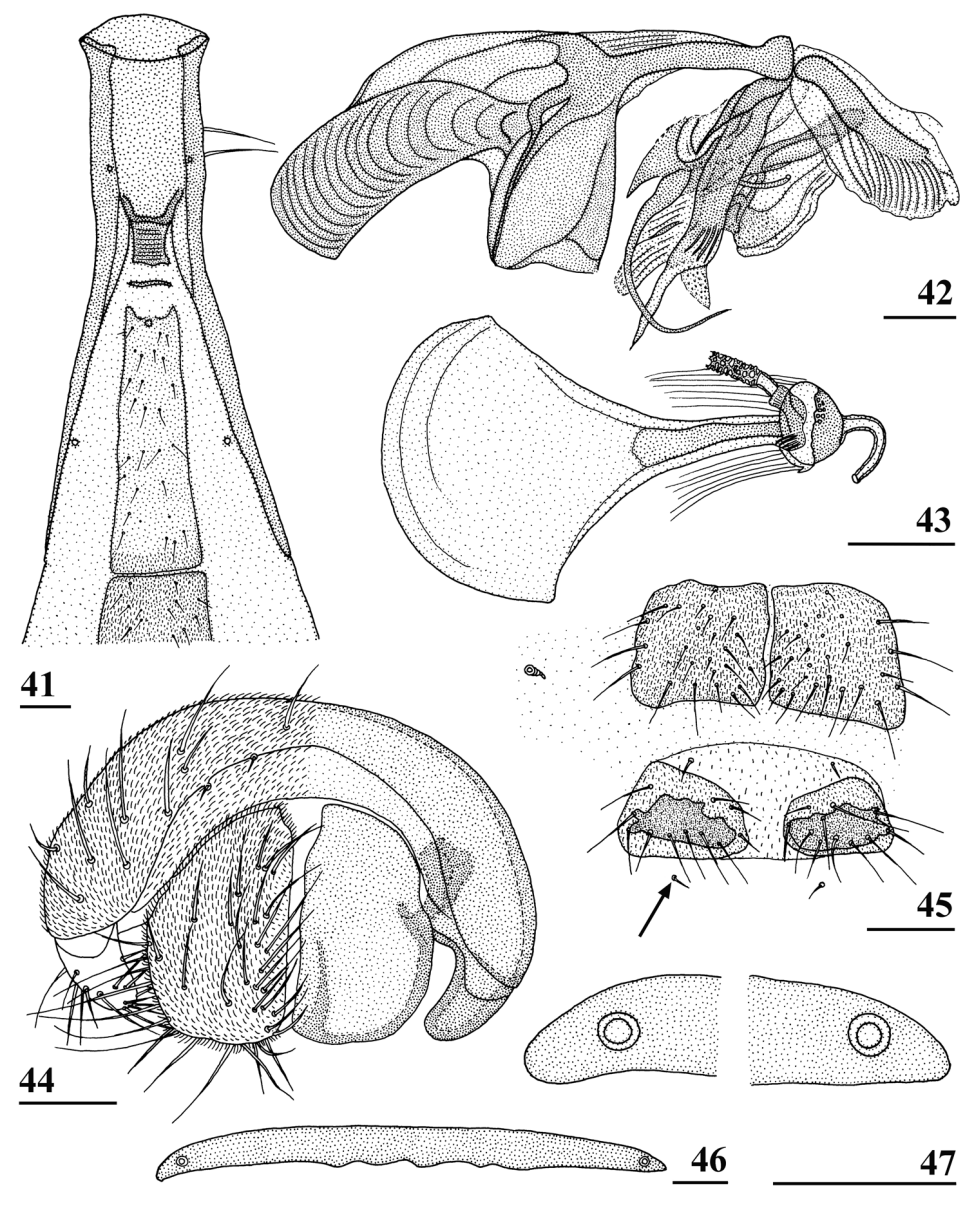
*Teleopsis neglecta* sp. nov., ♂, holotype, Pundaluoya **41** basal section of abdomen, ventral view **42** phallapodeme and aedeagus, lateral view **43** ejaculatory apodeme and sac **44** epandrium with surstyli and cerci, posterior view **45** sterna 4 and 5, ventral view (arrow indicating the anterior setulae of the otherwise absent sternum 6) **46** synsternum 7+8, ventral view **47** same, details of lateral sections. Scale bars: 0.2 mm (**41**); 0.1 mm (**42–47**).

***Preabdomen***. Terga 1, 2 and 3 reddish brown to black, other terga slightly darker; basal terga thinly pollinose, a glossy lateral spot on tergum 1 and basal half of tergum 2, laterally on tergum 3 a pair of more densely pollinose spots, tergum 4 glossy dorsally, tergum 5 densely pollinose, appearing pale grey on live photograph; seam between terga 2 and 3 not very distinct; sternum 1 and intersclerite blackish brown, other sterna brown, sternum 1 and most of sternum 2 glossy (Figs 40, 41), posterior edge of sternum 2 and other sterna thinly pollinose; basal three-quarters of sternum 1 fused to tergum 1 (Figs 40, 41), sternum 1 with typical U-shaped ridge posteriorly; spiracle 1 in tergum; intersclerite not clearly connected to sternum 2, sternum 2 hardly widening posteriorly, ratio length/width 3.8; sternum 3 a rectangular plate broadening posteriorly; sternum 4 consisting of two square sclerites, narrowly separated on the meson (Fig. 45).

***Male postabdomen*.** Sternum 5 represented by two sclerites with only the central sections strongly sclerotised; sternum 6 indiscernible, only the two characteristic anterior tiny setulae could be found (Fig. 45); synsternum 7+8 (Figs 46, 47) a symmetrical, very short, broad sclerite, hardly tapering laterally; spiracles 7 quite symmetrically in synsternum; epandrium (Fig. 44) broad, rounded, covered with microtrichia and about 12 pairs of setulae; surstyli articulate, slender, small and apically rounded in posterior view, in lateral view slightly curved, ratio length/width 2.5 (Figs 21, 44), glabrous, no microtrichia, on outer and apical side with about 18 setulae; surstyli connected to lateral side of cerci, not interconnected via processus longi; cerci large, broad, apical edges sclerotised (Fig. 44), ratio length/width 1.7, covered with microtrichia and especially along edges with setulae; phallapodeme (Fig. 42) strongly curving downward anteriorly, anterior arm broadening above the vane and 1.3× the posterior arm, posterior arm weakly bifurcated, vane broad; aedeagus a complicated open structure of sclerites and membranes (Fig. 42), rather long male genital process sticking out from apex; ejaculatory apodeme fan-shaped (Fig. 43).

#### Distribution.

*Teleopsis neglecta* sp. nov. is known from Sabaragamuwa Province, Southern Province and Central Province.

#### Etymology.

The specific epithet *neglecta* reflects the fact that, after collecting, it took 130 years for this species to be finally described (*neglecta*, ignored).

### Teleopsis sorora sp. nov.

Taxon classificationAnimaliaDipteraDiopsidae

2C42F565-756C-54FA-80FF-D8A61777AD81

http://zoobank.org/6C00A046-4E57-4B53-B3BB-1D486FCA0AF5

Figures 8 , 18 , 27 , 48–50 , 51 , 52–55 , 56–61 , 62 , 63

Teleopsis ferruginea : Feijen 1998: 55 (all specimens except for holotype of Diopsis ferruginea); Kotrba et al. 2013: 190, fig. 3f.

#### Type material.

***Holotype***, ♂ Sri Lanka, Kan. Dist. [Central Province, Kandy District], Udawattakele Sanct., [7°17'55"N, 80°38'32"E, ~ 600 m], 1–3.ix.1980, K.V. Krombein, P.B. Karunaratne, T. Wijesinhe, L. Jayawickrema, V. Gunawardane (USNM). ***Paratypes***: 3 ♀, 5 ♂, 1 ?sex, same data as holotype; 4 ♀, 6 ♂, Kandy, Udawattakele Sanct., Kan. Dist., 6–8.vi.1978, K. V. Krombein, P. B. Karunaratne, T. Wijesinhe, V. Kulasekare, L. Jayawickrema; 2 ♀, 3♂, Udawattakele Sanct., Kan. Dist., 8–11.ii.1979, K. V. Krombein, P. B. Karunaratne, T. Wijesinhe, S. Siriwardane, T. Gunawardane; 1 ♀, 3 ♂, Kandy Reservoir Jungle, Kan. Dist., [probably Darwin reservoir, 7°17'01"N, 80°38'18"E, 600 m], 10.ii.1979, K. V. Krombein, P. B. Karunaratne, T. Wijesinhe, S. Siriwardane, T. Gunawardane (all Krombein material in USNM with some specimens in RMNH); 1 ♂, C.P. [Central Province], Kandy, Roseneath [tea plantation?], [7°16'44"N, 80°38'19"E, ~ 655 m], 12.vii.1953, F. Keiser (NHMB); 1 ♂, C.P., Kandy, Roseneath, 11.viii.1953, F. Keiser (NHMB).

#### Notes.

Feijen (1998) included in the material studied as “*T. ferruginea*” the following specimens: 1 ♀, 1 ♂, Sri Lanka (NHMUK); 5 ♂, Sri Lanka, Dr. Thwaites, 67-25 (NHMUK); 2 ♀, 2 ♂, 3?, Sri Lanka, Weston Coll., NHMUK 1924-199; 1 ♀, 2 ♂, Peradeniya, Sri Lanka, 30.iv.1891, Lt Col. Yerbury (NHMUK); 2 ♂, Henaratgoda, Sri Lanka, i.1901 (NHMUK); 1 ♂, Suduganga, 10.ix.1919, R. Senior White (NHMUK). These specimens are also likely to represent *T. sorora* sp. nov., but this remains to be ascertained.

#### Additional material.

**Figures 48–50.**
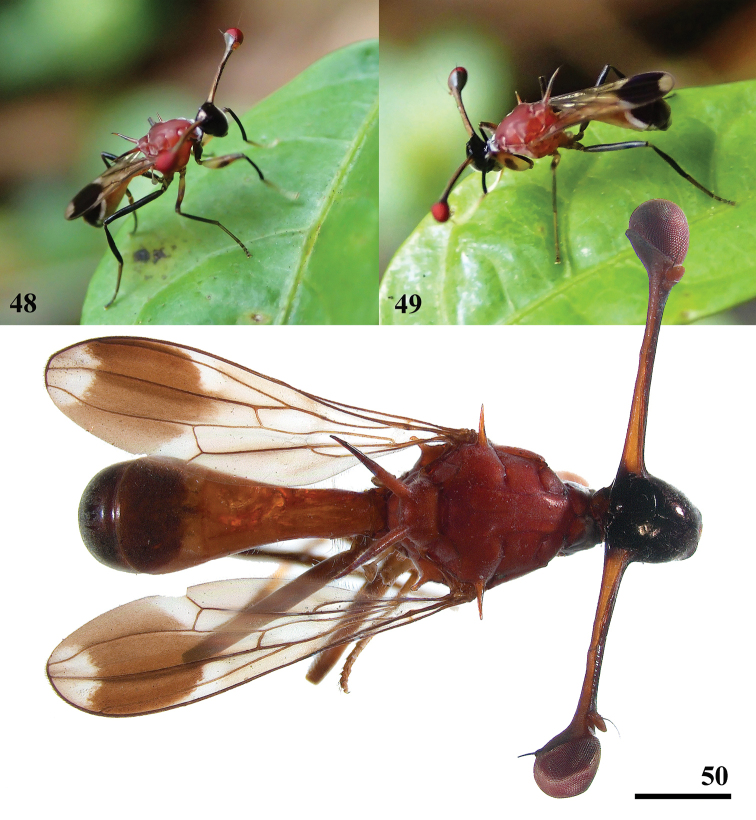
*Teleopsis sorora* sp. nov. **48–49** live photographs by Pieter D.H. Prins, Udawattakele (www.inaturalist.org/observations/36624976) **50** ♂, holotype, Udawattakele, photograph Cobi Feijen. Scale bar: 1 mm.

First set of photographs by Pieter D. H. Prins taken at Kandy, K. F. G. & G. Korale, Udawattakele Sanctuary, 7°18'11"N, 80°38'32"E, 22.xi.2010, 580 m, 23.xi.2010 and 12.xii.2010 (www.inaturalist.org/observations/35209123). Second set of photographs (www.inaturalist.org/observations/36624976) from same location and by the same photographer, 5.xii.2014 (Figs 48, 49).

#### Diagnosis.

*Teleopsis sorora* sp. nov. is the most colourful of all diopsids with its glossy black head, reddish legs, reddish thorax, reddish basal abdomen and black apical abdomen. Furthermore it can be recognized by its slender habitus, bareness, wing pattern (broad and curved dark preapical crossband and two very indistinct crossbands), small glabrous area of wing, setula-like inner vertical seta 0.6× the stalk diameter (usually broken off), medium-sized outer vertical seta 1.8× stalk diameter, small base of inner vertical seta, no facial teeth, blackish collar covered with dense white pollinosity, reddish brown, thinly pollinose scutum and scutellum, moderately curved scutellar spines, ratio scutellar spine/scutellum close to 3.0, moderately incrassate front femora (ratio length/width ~ 4.5) with around 48 (♀) to 50 (♂) tubercles, blackish terga 3–5 forming a circle, pair of pollinose spots on tergum 3, rectangular♀ sternum 6, almost completely divided ♀ sternum 7, ♀ spiracle 7 in membrane, rounded pentagonal subanal plate, rather elongate ♀ cerci, round spermathecae with 8–10 rounded protuberances, right ♂ spiracle 7 in synsternum 7+8, left spiracle 7 in lateral slit of synsternum, articulate and very small surstyli wider than long (ratio length/width 0.5) without microtrichia and with 10 setulae, large and rectangular ♂ cerci (ratio length/width 1.9), phallapodeme with broad anterior arm, strongly curving downward anteriorly, small eye span in female (79% of body length), medium-sized eye span in male (99% of body length), and low rate of sexual dimorphism with regard to eye span D = 0.82. *Teleopsis sorora* sp. nov. can be considered the sister species of *T. ferruginea* and forms part of the *T. ferruginea* species group.

#### Description.

The following description is partly based on the redescription of *Teleopsis ferruginea* by Feijen (1998: 55) for which specimens were used that are now placed in *Teleopsis sorora* sp. nov.

***Measurements*.** Body length ♀ 5.8 mm ± SE 0.2 (range 4.8–6.5, *N* = 9), ♂ 5.9 mm ± 0.1 (range 4.7–6.5, *N* = 20); eye span ♀ 4.6 mm ± 0.2 (range 3.8–5.3, *N* = 10), ♂ 5.9 mm ± 0.2 (range 4.1–7.3, *N* = 20); wing length ♀ 4.4 mm ± 0.2 (range 3.7–4.8, *N* = 8), ♂ 4.5 mm ± 0.1 (range 3.8–4.8, *N* = 17); length of scutellar spine ♀ 1.18 ± 0.03 (range 1.01–1.33, *N* = 9), ♂ 1.17 mm ± 0.02 (range 0.99–1.30, *N* = 19).

**Figure 51.**
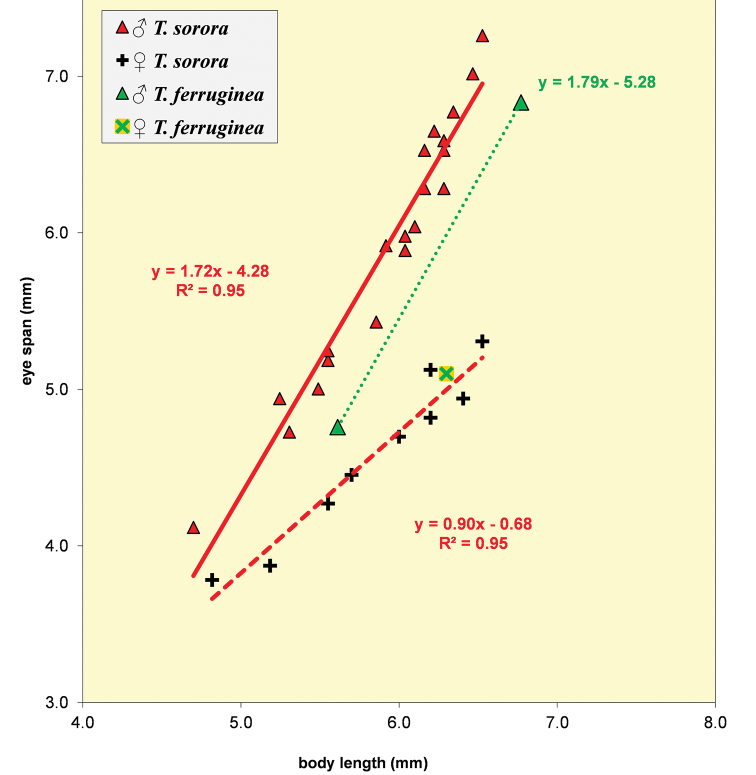
*Teleopsis sorora* sp. nov., eye span plotted against body length. For comparison, the three data points for *Teleopsis ferruginea* are also indicated.

***Head*.** Central part glossy black (Figs 27, 48–50), face laterally and ventrally covered with a typical, ‘woolly’ type of pollinosity, face and frons otherwise bare with only a few tiny pale setulae; frons (Fig. 27) very smooth with laterally at base of stalk a deep groove; arcuate groove narrow and concolourous; face flat and very smooth, facial sulcus indistinct, no facial teeth, lateroventral corners rounded; mouthparts greyish brown; eye span small in female (78.6 ± 0.9% of body length) and medium-sized in male (99.4 ± 1.5% of body length); a dimorphic species with a low rate of dimorphism D = 0.82 (Fig. 51); stalks yellowish brown, anteriorly and posteriorly with a blackish band, apices blackish pollinose; inner vertical seta usually appears minute, 0.1× the diameter of the eye stalk, in a single teneral specimen there is a small, setula-like inner vertical seta of 0.6× the stalk diameter (these slender inner vertical seta probably drop off early in the life of the fly); base of inner vertical seta small, just more than 0.2× the stalk diameter; outer vertical seta medium-sized, about 1.8× the stalk diameter, spinous.

***Thorax*.** Collar blackish brown, covered with dense white pollinosity, dorsoposterior edge brown and laterally a brown band; scutum, scutellum and scutellar spines brown, almost reddish brown, thinly pollinose (Figs 48–50), scutellar spines with darker central band; pleura brown, very thinly pollinose, katepisternum ventrally glossy, prosternum with black spot between front coxae; supra-alar spines (Fig. 50) glossy brown, almost 2½ x as long as pleurotergal spines, laterally directed, somewhat turned upward; scutellar spines moderately curving upward and outward, diverging under an angle of 75° (Fig. 50), ratio scutellar spine/scutellum in ♀ 2.79 ± 0.05 (*N* = 9) and in ♂ 2.98 ± 0.05 (*N* = 19), ratio scutellar spine/body length in ♀ and ♂ 0.20 ± 0.00 (*N* = 9, resp. 19); pleurotergal spines pollinose, medium-sized and blunt, posterolaterally directed; apical seta small, 0.15 ± 0.00% of length of scutellar spine (*N* = 21); very few tiny setulae on scutum, a few more on ventral thorax, scutellar spines with a few tiny setulae, no basal warts.

**Figures 52–55.**
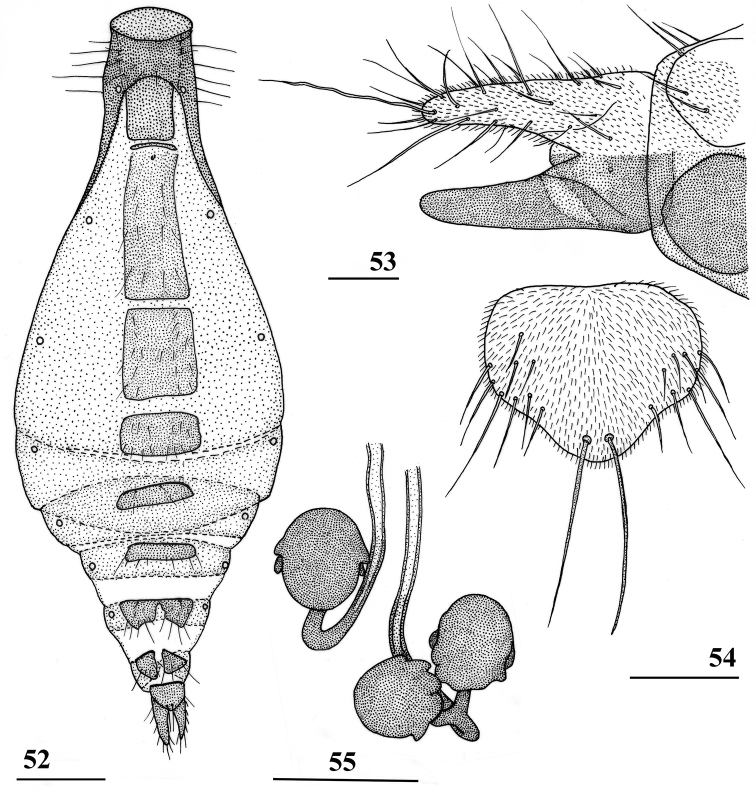
*Teleopsis sorora* sp. nov., ♀, paratype, Udawattakele **52** abdomen, ventral view **53** terga 8, 10 and cerci, dorsal view **54** subanal plate, ventral view **55** spermathecae. Scale bars: 0.5 mm (**52**); 0.1 mm (**53–55**).

***Wing*.** Irrorated with a dominant, dark, curved preapical band (Figs 8, 50) and, in addition, two very indistinct crossbands; apex (apical 12% of wing) infuscated, slightly less infuscated area centrally near preapical band; preapical band broad, curved, apically convex, proximally concave, uniformly very dark, broadly linked to central crossband in and around cell r4+5; two pale spots in between the central and preapical bands, one in cells r1 and r2+3, and one basally in cell m1; broad, but vague and irregular central crossband including crossveins r-m and dm-m, slightly darker around vein M4; narrow, indistinct and irregular basal band, connections to central band, giving two pale spots, one distally in cell br and one centrally in cell m4; cell r4+5 narrowing subapically; vein M4 from crossvein dm-m onward turning downward and reaching till three-fifth of the distance to the wing edge; glabrous basal areas including basal half of cell c, tiny spotbasally in cell r1, central quarter of cell br, basal third of cell bm+dm except for edges, and posterior half of cell cua. The two pale wing spots proximally of the dark preapical band clearly coincide with the pair of whitish, densely pollinose spots adjoining the black apical section of the abdomen (Figs 48–50, especially Fig. 49). The same phenomenon was observed in the sister species *T. ferruginea* (Feijen 1998). Coinciding wing spots and abdominal spots are a common phenomenon in several Diopsidae genera.

**Figures 56–61.**
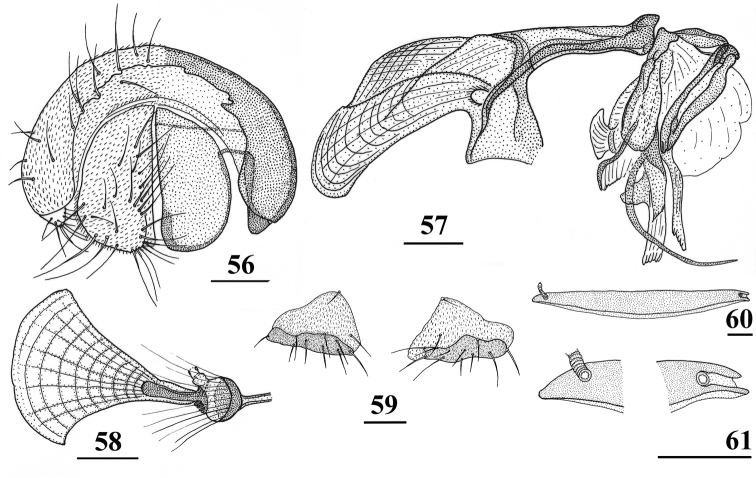
*Teleopsis sorora* sp. nov., ♂, paratype, Udawattakele **56** epandrium with surstyli and cerci, posterior view **57** phallapodeme and aedeagus, lateral view **58** ejaculatory apodeme and sac **59** sternum 5, ventral view **60** synsternum 7+8, ventral view **61** same, details of lateral sections. Scale bars: 0.1 mm.

***Legs***. Front leg with coxa glossy brown on outer side and dark brown pollinose on inner side, pale brown on anterior side, trochanter and femur pale brown, pollinose, femur with small dark spot basally on inner side and large black spot on distal two-thirds of outer side (Figs 48, 49), tibia glossy blackish brown, tarsus whitish except for brown base of metatarsus; mid leg brown with dark spots on femur and darker tibia; hind leg dark brown (almost black in the live specimens); femur 1 (Fig. 48) moderately incrassate with ratiolength/width in ♀ 4.5 ± 0.0 (*N* = 10) and in ♂ 4.6 ± 0.0 (*N* = 19), two rows of tubercles on distal three-quarters, inner row in ♀ with 25.7 ± 0.6 tubercles (range 22–30, *N* = 19) and in ♂ with 27.6 ± 0.4 tubercles (range 24–31, *N* = 26), outer row in ♀ with 21.9 ± 0.4 tubercles (range 19–25, *N* = 18) and in ♂ with 22.6 ± 0.4 tubercles (range 19–26, *N* = 28); legs with some setulae, ventral side of femur 1 densely covered with small setulae.

**Figure 62.**
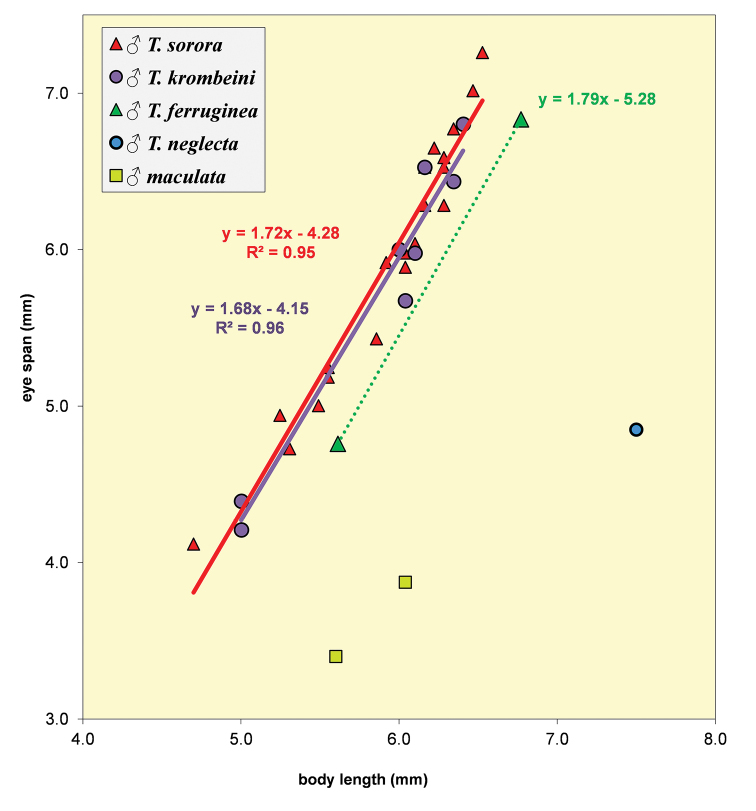
Eye span plotted against body length for the males of the five Sri Lankan *Teleopsis* showing the difference in allometric slopes between the dimorphic *T. sorora* sp. nov., *T. krombeini* and *T. ferruginea* and the few data points for the monomorphic *T. maculata* and *T. neglecta* sp. nov.

***Preabdomen***. Terga 1 and 2 and base of tergum 3 brown, remainder of tergum 3 and terga 4, 5 and 6 blackish brown, forming a black circle (Figs 48–50) which coincides with the dark preapical wing bands; two basal terga thinly pollinose except for glossy lateral parts, laterally on tergum 3 a pair of whitish, densely pollinose spots adjoining the blackish section, apical terga with dense, whitish pollinosity; seam between terga 2 and 3 very indistinct; sterna yellowish brown, sternum 1 and intersclerite brown, sternum 1 glossy,other sterna yellowish brown, pollinose; basal half of sternum 1 seamlessly fused to syntergum; spiracle 1 in sclerite; intersclerite not connected to sternum 2 (Fig. 52), sterna slightly broadening towards abdominal apex.

**Figure 63.**
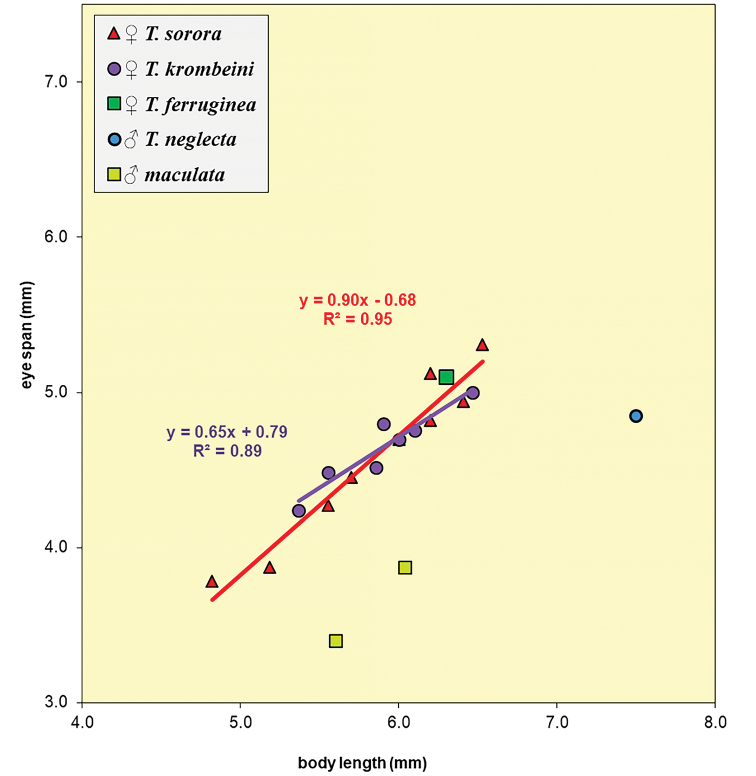
Eye span plotted against body length for the females of three dimorphic species *T. sorora* sp. nov., *T. krombeini* and *T. ferruginea* and the few data points for males of the monomorphic species *T. maculata* and *T. neglecta* sp. nov. Note the similarity in allometric slopes.

***Female postabdomen***. Deflexed; terga 6 and 7 single rectangular sclerites (Fig. 52); tergum 8 represented by two rounded sclerites (Fig. 53), sclerites anteriorly glabrous; tergum 10 ill-defined, with one pair of setulae; cerci rather elongate, ratio of length/width 3.6, covered with microtrichia and a number of setulae; sterna 5 and 6 single rectangular sclerites; sternum 7 posteriorly constricted medially, giving two sclerites joined anteriorly; spiracle 7 in membrane; sternum 8 represented by two triangular sclerites; subanal plate (Fig. 54) pentagonal with rounded corners, at apex a pair of large setulae; laterally about 8 pairs of small setulae, covered with microtrichia; spermathecae (Fig. 55) rounded with few rounded protuberances, ten in the single theca and eight each in the pair, heavily sclerotised; sclerotised ring of ventral vagina tapering towards one side.

**Figures 64–66.**
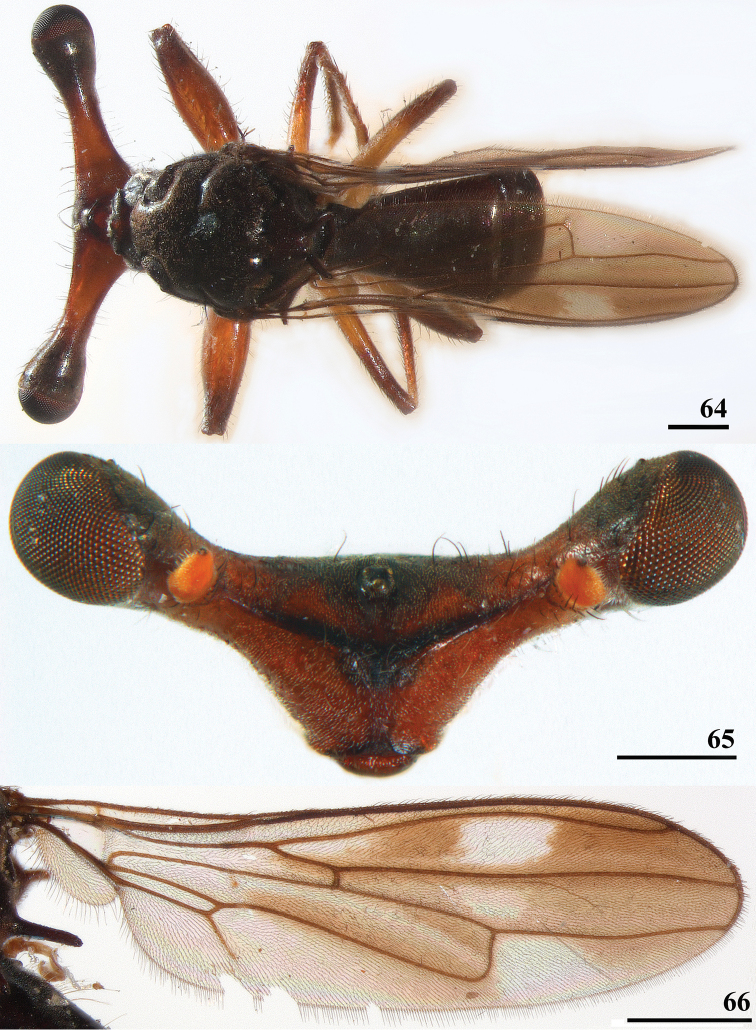
*Sphyracephala bipunctipennis*, paratypes, Sudu Ganga **64** ♂, habitus, dorsal view **65** ♀, head, anterior view (inner vertical seta absent, but outer vertical seta broken off) **66** ♂, wing. Scale bars: 0.5 mm.

***Male postabdomen***. Sternum 4 a single rectangular sclerite; sternum 5 consisting of two small strongly sclerotised sclerites with anteriorly vaguely sclerotised sections (Fig. 59); sternum 6 indiscernible; synsternum 7+8 without sclerotised connection to anterior sclerites of epandrium; right spiracle 7 in anterior edge of synsternum, left spiracle 7 in slit in lateral tip of synsternum (Figs 60, 61); epandrium (Fig. 56) rounded, covered with microtrichia and about 14 pairs of setulae; surstyli articulate, very small, apically rounded, in lateral view asymmetrical (one corner acute and the other one rectangular), wider than long, ratio length/width 0.5 (Fig. 18), no microtrichia, apically 10 setulae, setulae about as long as surstylus; surstyli just connected to lateral side of cerci, not interconnected via processus longi; cerci large, broad, flat, quite rectangular with rounded corners, equal width along most of its length, ratio length/width 1.9, covered with microtrichia and especially along edges with setulae; phallapodeme (Fig. 57) with broad anterior arm, abruptly narrowing anteriorly and strongly curving downward, anterior arm slightly longer than posterior arm; aedeagus (Fig. 57) with rather long male genital process sticking out from apex; ejaculatory apodeme fan-shaped (Fig. 58), ejaculatory sac relatively small.

**Figures 67–69.**
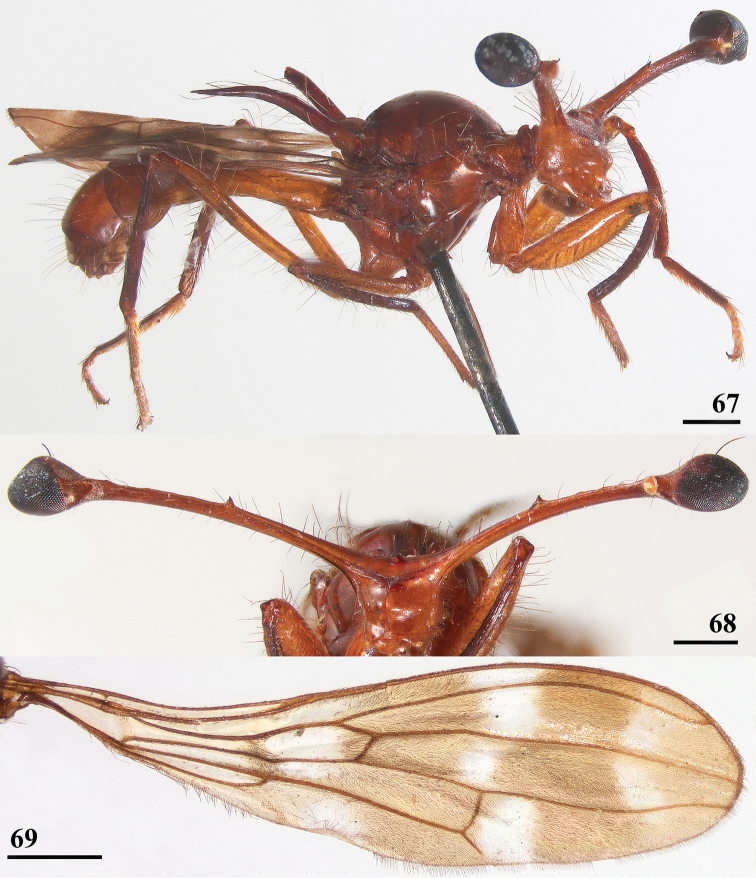
*Cyrtodiopsis* sp., Morin? **67** ♀, habitus, lateral view **68** ♂, head, anterior view (inner vertical seta broken off) **69** ♀, wing. Scale bars: 0.5 mm.

#### Distribution.

The specimens of the type series are from three neighbouring locations in Kandy: Udawattakele Sanctuary, Darwin Reservoir and Roseneath. If the NHMUK specimens also represent *T. sorora* sp. nov., this would extend the known distribution to the surroundings of Colombo and the wider surroundings of Kandy.

**Figures 70–72.**
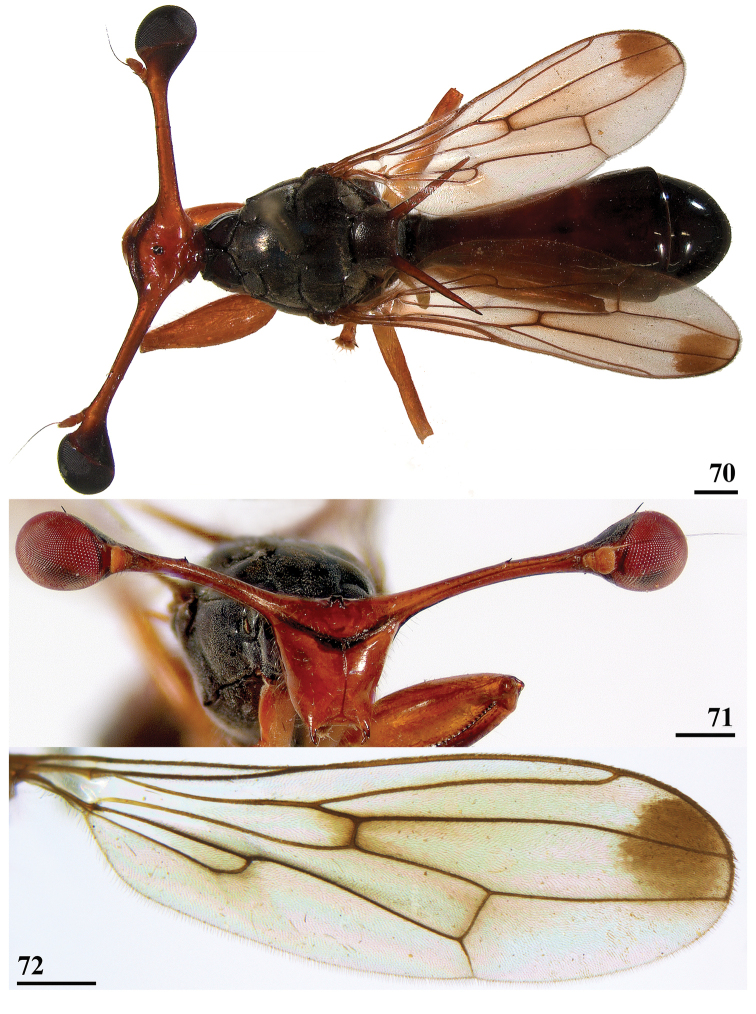
*Diopsis* sp., Mahiyangana **70** ♂, habitus, dorsal view **71** ♂, head, anterior view **72** ♀, wing. Scale bars: 0.5 mm.

#### Etymology.

This new species is considered the sister species of *Teleopsis ferruginea*, hence the name *sorora* (sister).

**Figure 73.**
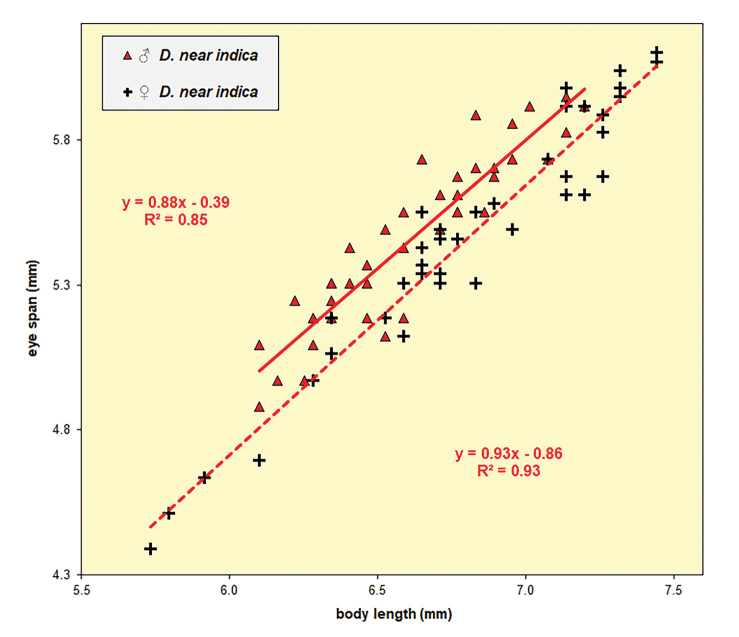
Diopsis nr indica from Sri Lanka, eye span plotted against body length. The graph is based on measurements for 40 ♀ and 40 ♂ and demonstrates an unequivocally homomorphic species.

## Discussion

### The biogeographic range of *Teleopsis*

Feijen and Feijen (2011) discussed the biogeographic range of the genus *Teleopsis*. They indicated *Teleopsis* s. s. as a purely Oriental genus with species occurring in India, ? Myanmar, Sri Lanka, Indonesia (Sumatra, Java, Bali and Borneo only), Malaysia, Brunei,Thailand, China (only Hainan), and the Philippines. For the second Diopsidae genus with supra-alar spines, *Megalabops* Frey, they gave a more northern distribution with species in occurring in Nepal, Northern India, Myanmar, West Malaysia, Thailand, Cambodia, Vietnam, and China (mainland and Taiwan). To the range for *Teleopsis* can now be added Vietnam and Southern Mainland China (Yunnan), while to the range of *Megalabops* can be added Bhutan (see Feijen and Feijen 2019) and Laos.

Feijen and Feijen (2019) discussed the *Teleopsis* species groups in India and Sri Lanka. The Indian and Sri Lankan *Teleopsis* form isolated groups in their genus from morphological as well as geographical point of view. In India, only the *Teleopsis sykesii* species group occurs with its two species distributed in Western India. From Eastern India, *Teleopsis* are not known, so the *T. sykesii* group forms an isolated group. The *Teleopsis* species geographically closest to the *T. sykesii* group are found in Sri Lanka. They belong to the equally isolated *T. ferruginea* species group. Otherwise the nearest *Teleopsis* members are found in Thailand and Peninsular Malaysia. The two *T. sykesii* records for Myanmar are very doubtful (Feijen and Feijen 2011). Molecular data for India and Sri Lankan *Teleopsis* are still lacking, but a close relationship between the two species groups appears unlikely.

### The *Teleopsis ferruginea* species group

For the phylogenetic position of *Teleopsis* within the Diopsidae can be referred to Feijen et al. (2018a). *Teleopsis* forms part of the *Teleopsis* genus group, characterised by irrorated wings. Within *Teleopsis*, the species of the *T. ferruginea* group can be characterised by their bareness, small to medium-sized inner vertical seta (0.5–1.5× stalk diameter), tiny to small base of inner vertical seta (0.1–0.5× stalk diameter), absence of facial teeth, moderately incrassate to incrassate front femora (mean ratio length/width varying from 3.8–4.9), male sternum 5 consisting of two sclerites, indiscernible male sternum 6, small and articulating surstyli with 10–25 setulae and without microtrichia, and ribbon-like male synsternum with 7^th^ spiracles in sclerite or in lateral slit of sclerite. Female genitalia have only been described for *T. krombeini* and *T. sorora* sp. nov. Based on only these two species, additional character states of the *T. ferruginea* group might be: ♀ tergum 7 and sternum 7 unconnected, ♀ spiracles 7 in membrane, and spermathecae with very short tubercles.

Within the species group, *T. ferruginea*, *T. sorora* sp. nov., and *T. krombeini* form a subgroup based on similarities in shape and setulae of the surstylus, the lateral slit in the male synsternum accommodating left spiracle 7, the sexual dimorphism with regard to eye span and the similar allometric slope for male eye span on body length. *Teleopsis ferruginea* and *T. sorora* sp. nov. are obvious sister species based on shape and colouration of central head, colour pattern of scutum and dorsal abdomen and male genitalia. The sexually monomorphic *T. neglecta* sp. nov. and *T. maculata* stand separate from the three dimorphic species, given also that in both species the left male spiracle 7 is located in sclerite. However, as indicated above, more information is required to determine the relationships between *T. neglecta* sp. nov. and *T. maculata* and with the group of dimorphic species.

### Dimorphism and monomorphism in the Sri Lankan *Teleopsis*

Baker and Wilkinson (2001), comparing allometric data with a phylogenetic tree based on molecular analyses, concluded that for the Diopsidae “Sexual dimorphism in eye span has evolved independently at least four times in the family ...”. Later, Feijen and Feijen (2014) and F.A.A. Feijen (pers. obs.) found that there were, at least, eight cases of independent development of sexual dimorphism with regard to eye span within the Diopsidae. This concerned three cases in the Sphyracephalinae, one in the *Diasemopsis* genus group, at least three in the *Teleopsis* genus group (the irrorated wings group) and one in the genus *Diopsis*. The dimorphic Malagasy *Diopsis nigrosicus*, distantly related to the monomorphic *Diopsis ichneumonea* species group of African Mainland, provided the ninth case (Feijen et al. 2018b). The five Sri Lankan *Teleopsis* species can, based on morphological criteria, be assumed to belong to the monophyletic *Teleopsis ferruginea* species group. Within this species group there are three distinctly dimorphic species: *T. ferruginea*, *T. krombeini*, and *T. sorora* sp. nov. For the other two species, *T. maculata* and *T. neglecta* sp. nov., only a few specimens are available, but they can, in all likelihood, be assumed to represent monomorphic species. As such the number of cases of independent development of sexual dimorphism with regard to eye span now comes to ten.

### Key to the Diopsidae species of Sri Lanka

**Table:** 

1	Arista tripartite, alula present, vein CuA+CuP extending past cell cua (Fig. 66), syntergum including terga 1–2, apical scutellar seta several times longer than scutellar spine	***Sphyracephala bipunctipennis* (Senior-White, 1922)**
–	Arista bipartite, alula absent, vein CuA+CuP not extending past cell cua (Figs 69, 72), syntergum including terga 1–3, apical scutellar seta smaller than the length of the scutellar spine or absent	**Diopsinae 2**
2	Scutellar spine almost straight, apical bristle absent, wing with dark and round apical wing spot and vague central infuscation especially around crossvein r-m (Fig. 72), scutum uniformly black	**Diopsis near indica**
–	Scutellar spine strongly curved, apical bristle present (often broken off), irrorated wings with dark crossbands and pale spots (Figs 7–11, 69), scutum reddish or brown	**3**
3	No supra-alar spines, covered with long setulae, especially head and legs (Fig. 67), inner vertical seta long (≥3× stalk diameter), female tergum 7 and sternum 7 forming a complete ring	**Cyrtodiopsis near dalmanni**
–	Supra-alar spines present, almost bare (only some tiny setulae) (Figs 1, 22, 23), inner vertical seta tiny to medium-sized (≤1.5× stalk diameter), female tergum 7 and sternum 7 not forming a complete ring	**4**
4	Ratio eye span/body length in males < 0.7 (Fig. 33), sexual monomorphism with regard to eye span, left male spiracle 7 in synsternum	**5**
–	Ratio eye span/body length in males > 0.85, on average ~1.0 (Fig. 22), sexual dimorphism with regard to eye span, left male spiracle 7 in lateral slit of synsternum	**6**
5	Central head and collar glossy black (Fig. 33), wing with dark, V-shaped preapical crossband and distinct apical spot (Fig. 10), dorsal abdomen with posterolateral pale spots on first five terga, pale spots on tergum 3 covered with dense pollinosity, terga 4 and 5 pollinose, femur 1 with two distinct brown spots on inner side, surstylus broad and short (ratio length/width 0.6, Fig. 20)	***Teleopsis maculata* Feijen, 1998**
–	Central head yellowish brown and thinly pollinose (Figs 26, 37), collar reddish brown, wing with three dark crossbands strongly interconnected giving four distinct pale spots (Fig. 11), no lateral pale spots on first five terga, tergum 3 with a pair of densely pollinose spots, tergum 4 glossy dorsally, tergum 5 densely pollinose, femur 1 with very vague brown stripe on inner side, surstylus narrow and long (ratio length/width 2.5, Fig. 21)	***Teleopsis neglecta* sp. nov.**
6	Central head brown, concolorous with stalks (Fig. 24), upper half of face more pronounced, face tapering ventrally, inner vertical seta spinous, scutum brown with posterior half glossy, wing with central and preapical crossbands equal in width (Fig. 9), dorsal abdomen yellowish brown basally and dark brown more apically, surstyli relatively large with 25 setulae (Fig. 19)	***Teleopsis krombeini* Feijen, 1998**
–	Central head black, stalks brown, face smooth and rounded (Fig. 2), inner vertical seta a thin setula (usually broken off), scutum reddish brown and thinly pollinose, wing with broad dominant preapical crossband (Figs 7, 8), dorsal abdomen reddish brown with terga 3 and 4 forming a black circle, surstyli very small with 10 setulae (Figs 17, 18)	**7**
7	Wing with three distinct crossbands, preapical crossband straight (Fig. 7), front femur pollinose with vague darker spot on outer side, very small surstyli with ratio length/width 1.0 (Fig. 17), male cerci widest subapically (ratio length/width 1.6), phallapodeme quite straight, anterior arm slightly curving downward anteriorly	***Teleopsis ferruginea* (Röder, 1893)**
–	Wing with one dominant, dark, curved preapical crossband and two indistinct crossbands (Fig. 8), front femur with small dark spot basally on inner side and large black spot on distal two-thirds of outer side, very small surstyli with ratio length/width 0.5 (Fig. 18), rectangular male cerci (ratio length/width 1.9), phallapodeme with broad anterior arm strongly curving downward anteriorly	***Teleopsis sorora* sp. nov.**

### Other Diopsidae in Sri Lanka

**Notes.** The three other Diopsidae known from Sri Lanka are *Sphyracephala bipunctipennis*, *Cyrtodiopsis* sp. and *Diopsis* sp. For descriptions of the latter two species, large scale revisions will be required for, respectively, the *Cyrtodiopsis dalmanni* species group and the *Diopsis indica* species group. Here, the collecting data are given for the three species concerned. For all three species illustrations are provided for the habitus (Figs 64, 67, 70), the anterior view of the head (Figs 65, 68, 71) and the wing (Figs 66, 69, 72). Asa very large sample of flies was available for the *Diopsis* sp., its monomorphism with regard to eye span, will briefly be discussed.

#### *Sphyracephala* Say, 1828

##### Sphyracephala bipunctipennis

Taxon classificationAnimaliaDipteraDiopsidae

(Senior-White, 1922)

E53A9142-E109-51C5-999D-B77F56F46758

Figures 64–66

Teleopsis bipunctipennis Senior-White, 1922: 165, pl. 13, fig. 1; Descamps 1957: 19; Steyskal 1972: 11.
Pseudodiopsis bipunctipennis (Senior-White): Shillito 1940: 150; Steyskal 1977: 35.
Sphyracephala bipunctipennis (Senior-White): Feijen 1989: 67; Feijen 1998: 50; Feijen and Feijen 2019: 39.

###### Type material.

***Holotype***, ♂ Sri Lanka, [Central Province] Suduganga [Sudu Ganga] river, Indiganga, on leaves of Liliacrans (sic!) plant, 10.viii.1919 (NHMUK). ***Paratypes***: 7 ♀, 4 ♂, same data as holotype (NHMUK).

###### Distribution.

India (Tamil Nadu), Sri Lanka (Central Province). Except for the 1919 type series, no other specimens are known from Sri Lanka.

#### *Cyrtodiopsis* Frey, 1928

##### Cyrtodiopsis

Taxon classificationAnimaliaDipteraDiopsidae

sp.

4E074B85-D2F5-5E30-9E73-43CAF36E496A

Figs 67–69

###### Material examined.

2 ♀, 1 ♂, Ceylon [Sri Lanka], Morin, 1914 (ZMUO). The label information is very limited. It is not clear whether “Morin” is a locality name or the name of the collector. The only reference to Morin for Sri Lanka is a “Morin Inn” in Negombo.

###### Notes.

This species forms part of the *Cyrtodiopsis dalmanni* species group. The presence of *Cyrtodiopsis* in Sri Lanka would be rather surprising, given that the nearest relatives in the *C. dalmanni* group occur in Malaya and Indonesia. The geographically closest *Cyrtodiopsis* is *C. whitei* Curran from north-eastern India. However, that species belongs to a different species group. The Sri Lankan record certainly requires confirmation to exclude the possibility of mislabelling.

Although many non-taxonomic papers have been written about “*Cyrtodiopsis dalmanni*”, the taxonomy of this species and the *C. dalmanni* species group still requires a full-scale taxonomic revision. This species group can be characterized by the many, long setulae covering the body, the wing pattern with three pale spots in between the central and preapical crossbands and the peculiar peg and hollow modification on the male front leg, the peg located basally on the tibia and the hollow distally on the femur. This leg modification is also referred to as “nutcracker” and can be found in all males, except for small ones. For illustrations of this modification can be referred to Földvári et al. (2019: fig. 4).

#### *Diopsis* Linnaeus, 1775

##### Diopsis

Taxon classificationAnimaliaDipteraDiopsidae

sp.

B27BDDD7-63C3-508C-9D04-81D6F02D26D6

## Supplementary Material

XML Treatment for Teleopsis

XML Treatment for Teleopsis ferruginea

XML Treatment for Teleopsis krombeini

XML Treatment for Teleopsis maculata

XML Treatment for Teleopsis neglecta

XML Treatment for Teleopsis sorora

XML Treatment for Sphyracephala bipunctipennis

XML Treatment for Cyrtodiopsis

XML Treatment for Diopsis
